# Selective Translation of Maternal mRNA by eIF4E1B Controls Oocyte to Embryo Transition

**DOI:** 10.1002/advs.202205500

**Published:** 2023-02-08

**Authors:** Jing Guo, Hailian Zhao, Jue Zhang, Xiangjiang Lv, Shen Zhang, Ruibao Su, Wei Zheng, Jing Dai, Fei Meng, Fei Gong, Guangxiu Lu, Yuanchao Xue, Ge Lin

**Affiliations:** ^1^ Clinical Research Center for Reproduction and Genetics in Hunan Province Reproductive and Genetic Hospital of CITIC‐Xiangya Changsha 410078 P. R. China; ^2^ Key Laboratory of RNA Biology Institute of Biophysics Chinese Academy of Sciences Beijing 100101 P. R. China; ^3^ University of Chinese Academy of Sciences Beijing 100049 P. R. China; ^4^ Laboratory of Reproductive and Stem Cell Engineering NHC Key Laboratory of Human Stem Cell and Reproductive Engineering Central South University Changsha 410078 P. R. China

**Keywords:** eIF4E1B, infertility, oocyte to embryo transition, RNA‐binding protein, translation

## Abstract

Maternal messenger ribonucleic acids (mRNAs) are driven by a highly orchestrated scheme of recruitment to polysomes and translational activation. However, selecting and regulating individual mRNAs for the translation from a competitive pool of mRNAs are little‐known processes. This research shows that the maternal eukaryotic translation initiation factor *4e1b* (*Eif4e1b*) expresses during the oocyte‐to‐embryo transition (OET), and maternal deletion of *Eif4e1b* leads to multiple defects concerning oogenesis and embryonic developmental competence during OET. The linear amplification of complementary deoxyribonucleic acid (cDNA) ends, and sequencing (LACE‐seq) is used to identify the distinct subset of mRNA and its CG‐rich binding sites within the 5′ untranslated region (UTR) targeted by eIF4E1B. The proteomics analyses indicate that eIF4E1B‐specific bound genes show stronger downregulation at the protein level, which further verify a group of proteins that plays a crucial role in oocyte maturation and embryonic developmental competence is insufficiently synthesized in *Eif4e1b*‐cKO oocytes during OET. Moreover, the biochemical results in vitro are combined to further confirm the maternal‐specific translation activation model assembled by eIF4E1B and 3′UTR‐associated mRNA binding proteins. The findings demonstrate the indispensability of eIF4E1B for selective translation activation in mammalian oocytes and provide a potential network regulated by eIF4E1B in OET.

## Graphical abstract

A new mechanism of selecting and regulating individual mRNAs for the initiation of translation from a competitive pool of mRNAs in mammalian oocytes during the process of oocyte to embryo transition: eIF4E1B interacts with 3′UTR‐associated RBPs to activate translation by targeting the GC‐rich elements in 5′ UTR of particular groups of transcriptome.

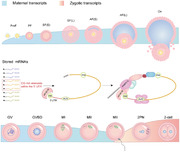

## 1 Introduction

In the process of oocyte‐to‐embryo transition (OET), maternal messenger ribonucleic acids (mRNAs) are actively transcribed during oogenesis and cease at the final stages of oocyte maturation, followed by zygotic genome activation (ZGA) after fertilization.^[^ ^1^, ^2^, ^3^ ^]^ Besides, a series of dramatic changes, including germinal vesicle breakdown (GVBD), spindle formation, fertilization, and maternal‐to‐zygotic transition (MZT), rely on previously transcribed maternal mRNAs that are driven by a highly orchestrated scheme of selective translation.^[^ ^3^, ^4^, ^5^ ^]^ This phenomenon, known as the key regulation process of post‐transcriptional control, has served as a major mystery in understanding OET.

As mRNAs covered by protein factors form mRNA–protein complexes (mRNPs), whose translation efficiency is affected by at least five elements: 5′cap, 5′ untranslated region (UTR), 3′ poly(A) tail and 3′UTR as well as its binding proteins.^[^ ^6^, ^7^, ^8^, ^9^ ^]^ In the process of cap‐dependent translation initiation, 5′cap structure is particularly recognized by eukaryotic translation initiation factor 4E (eIF4E) in association with eIF4G protein to constitute the eIF4F complex. eIF4G offers a crucial link to the 3′ poly(A) tail and poly(A) binding proteins (PABPs) interacting with this poly(A) tail, thereby forming a closed loop for efficient translation.^[^ ^10^ ^]^

It is well established that cytoplasmic polyadenylation activates dormant mRNAs in mammalian oocytes via cytoplasmic polyadenylation elements (CPEs) within their 3′ UTR and its cognate binding protein CPEB1.^[^ ^11^, ^12^ ^]^ mRNAs with short poly(A) tails are regulated by deleted in azoospermia‐like (DAZL) family proteins through recruiting  PABPs.^[^ ^13^, ^14^, ^15^, ^16^ ^]^ Regardless of the proposed mechanism, eukaryotic 5′UTR is of critical importance for the control of translation efficiency. Eukaryotic initiation factors (eIFs) and particular sequence motifs play an essential role in translation initiation at 7‐methylguanosine (m7G) mRNA caps.^[^ ^10^, ^17^, ^18^, ^19^ ^]^ It has been identified that a group of eIF4E‐sensitive mRNAs featuring a 5′ terminal oligopyrimidine (TOP) motif necessary for spindle assembly and chromosome alignment and segregation were temporal and spatial translation controlled during the meiotic process of mammalian oocytes via the activity of the mammalian target of rapamycin (mTOR)‐eIF4F pathway.^[^ ^20^ ^]^ It is considered that cap‐binding protein eIF4E is the most important factor in the eIF4F complex.^[^ ^21^ ^]^ To be specific, studies on *C. elegans* and *Xenopus* have elucidated that the individual isoforms of eIF4E have distinct developmental functions by tethering to a unique subset of mRNAs by means of RNP complexes in a tissue‐specific manner.^[^ ^22^, ^23^, ^24^, ^25^ ^]^

Nevertheless, the regulation of translational activation is quite complex and involves not only one aspect. In recent years, great progress has been made in the translation during mammalian OET, little is known of the oocytes selectively determine the group of mRNAs activated for translation.^[^ ^26^, ^27^, ^28^, ^29^, ^30^, ^31^ ^]^

Here, the effect and underlying mechanism of an oocyte‐specific eIF4E isoform, eukaryotic translation initiation factor 4e1b (eIF4E1B), was addressed by deleting eIF4E1B in developing oocytes. The genetic ablation of *Eif4e1b* resulted in a developmental arrest in the two‐cell phase of embryogenesis eventually. The unique mRNA network targeted by eIF4E1B was identified by using a low‐input crosslinking immunoprecipitation coupled with high‐throughput sequencing named LACE‐seq previously developed.^[^ ^32^ ^]^ The GC‐rich elements in 5′ UTR of groups of identified transcriptome contribute to the eIF4E1B binding preference. The unique mRNA network regulated by eIF4E1B was further verified by proteomics analyses in *Eif4e1b* deleted oocytes. In addition, eIF4E1B function together with a subgroup of RNA binding proteins (RBPs) got involved in promoting translational activation. Overall, it was unveiled that eIF4E1B has important functions in the selective translation activation of its particular target mRNAs within the cytoplasm to instruct oocyte maturation and developmental competence in mammals during OET.

## 2 Results

### 2.1 Expression of eIF4E1B During the OET

**Figure 1:**
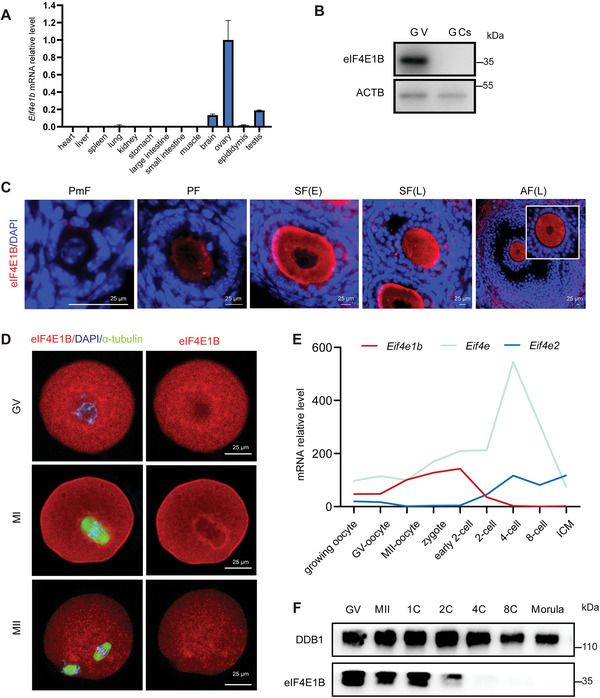
Expression of eIF4E1B during the OET. A) Quantitative RT‐PCR findings indicating relative expression levels of mouse *Eif4e1b* within different tissues. B) Western blotting findings of eIF4E1B protein levels in GV oocytes and granulosa cells in the ovary. C) Immunofluorescent images of ovary sections that are stained with anti‐eIF4E1B antibody (red) and DAPI (blue) for primordial, primary, secondary (early), secondary (late), and antral follicular stages. Scale bar, 25 µm. D) Fluorescence microscopy showing endogenous eIF4E1B expression at different stages of oocytes. Scale bar, 25 µm. E) The FPKM of detected eIF4Es in RNA‐seq data (GSE71434) at each developmental stage of mouse oocytes and early embryos. F) Western blotting findings of eIF4E1B protein levels within GV and MII oocytes, 1‐cell (1C), 2‐cell (2C), 4‐cell (4C), 8‐cell (8C), and morula stage embryos. Every lane was loaded with the total proteins from a total of 50 oocytes or embryos. DDB1 was blotted as the control of loading. Abbreviations: PmF, Primordial follicles; PF, Primary follicles; SF(E), Early secondary follicles; SF(L), Late secondary follicles; AF(L), Large antral follicles; GV, germinal vesicle oocyte; MI, metaphase I; MII, metaphase II‐arrested oocyte; GCs, granulosa cells.

Compared with other eIF4Es like *Eif4e* and *Eif4e2*, quantitative analyses by RT‐qPCR using the total RNA isolated from tissue lysates suggested that *Eif4e1b* was highly expressed in ovary than in other organs (**Figure** **1**A; Figure S1A,B, Supporting Information). Western blotting (WB) using a rabbit polyclonal antibody specific to 1–244aa of eIF4E1B showed that eIF4E1B expressed in oocytes and undetectable in granulosa cells of the ovary, indicating a specific expression in germ cells (Figure 1B). Immunohistochemistry (IHC) confirmed that eIF4E1B at the cellular localization during oogenesis had very low protein levels in the oocytes of primordial follicles and demonstrated elevated expression after the activation of the primordial follicle (Figure 1C). Germinal vesicle (GV), metaphase I (MI), and metaphase II (MII) oocytes were gathered to detect the expression of eIF4E1B which was distributed across the cytoplasm of GV and MII oocytes and locally concentrated around chromosomes and the perispindular area at MI stage (Figure 1D). RNA‐seq datasets (GSE71434) in mouse oocytes and early embryos showed that murine *Eif4e1b* mRNA was transiently decreased after 2‐cell stages, in comparison with other *Eif4e*s (Figure 1E).^[^ ^33^ ^]^ The in vivo expression of eIF4E1B at different stages of oocytes and early embryos was detected by WB. Consistently, eIF4E1B was detected in GV, MII oocytes, and 1‐cell to 2‐cell stages embryos. However, the respective amounts of eIF4E1B decreased in two‐cell embryos and were undetectable in four‐cell ones (Figure 1F). Their accumulation during the growth and degradation of oocytes after the two‐cell stage indicated the importance of eIF4E1B in the process of OET.

### 2.2 Defective Development of the Follicles in eIF4E1B‐Deficient Mice

**Figure 2:**
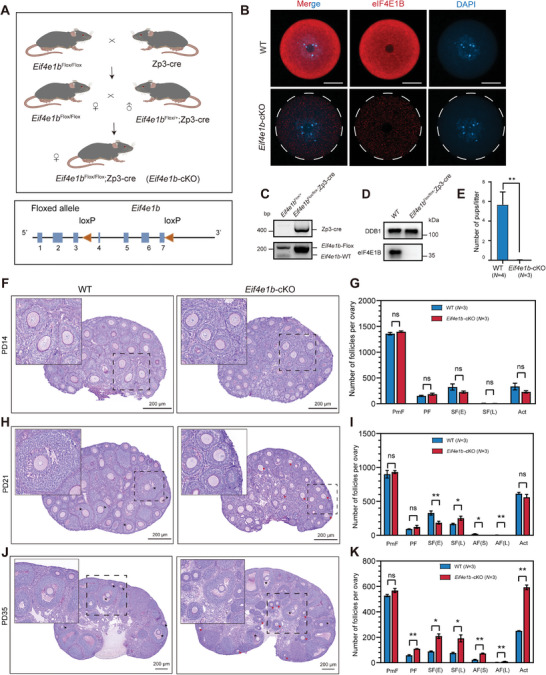
Defective development of the follicles in eIF4E1B‐deficient mice. A) *Eif4e1b* conditional knockout (*Eif4e1b*‐cKO or *Eif4e1b* ^loxP/loxP^; Zp3−cre) strategy within mouse oocytes schematic. The red triangles refer to the two loxP sites which are inserted at regions from exon 4 to 7. B) Immunofluorescent staining for anti‐eIF4E1B antibody (red) and DAPI (blue) for GV oocytes from WT and *Eif4e1b*‐cKO females. Scale bar, 25 µm. C) The agarose gel at the bottom panel refers to the genotyping result with two primers (F+R) that flank both sides of a certain loxP site. The Zp3‐cre expression is on the upper panel. D) Western blotting analysis of eIF4E1B expression and internal control DDB1 within WT and *Eif4e1b*‐cKO GV oocytes. E) Number of pups born by WT and *Eif4e1b*‐cKO females after an 8‐month fertility test. F–K) Morphologies and quantification of ovarian follicles in WT and *Eif4e1b*‐cKO mice: representative follicles and the number of each stage of follicles in F,G) PD14, H,I) PD21, J,K) PD35 *Eif4e1b*‐cKO are indicated in the figures. The top left corner square serves as a magnified image of the respective follicles (with an asterisk, showing antral follicles; with a black arrow, showing late secondary follicles in the WT group; and with a red arrow, showing late secondary follicles in *Eif4e1b*‐cKO group). Abbreviations: PmF, Primordial follicles; PF, Primary follicles; SF(E), Early secondary follicles; SF(L), Late secondary follicles; AF(S), Small antral follicles; AF(L), Large antral follicles; Act, activated follicles. *n*, number of cells; *N*, number of the experiment performed.

To investigate the in vivo function of maternal eIF4E1B, *Eif4e1b*‐deficient mice were generated with targeted deletion exons 4–7 of the *Eif4e1b* gene in oocytes by crossing male transgenic (Tg) mice [Tg(Zp3‐cre)93Knw mice] expressing Cre recombinase with female mice carrying the conditional allele of *Eif4e1b* (*Eif4e1^bem1(flox)^*) at early growth stages (**Figure** **2**A). Zp3‐Cre mediated cKO mice (*Eif4e1b*‐cKO) were genotyped and confirmed by genomic PCR and WB (Figure 2C,D). The specificity of the eIF4E1B antibody was confirmed on oocytes from *Eif4e1b* conditional knockout mice (Figure S1C, Supporting Information). Two eIF4E1B‐related bands with similar molecular weight (≈35 kDa) were detected in wild‐type (WT) oocytes. However, it is worth noting that these two bands were not detected in *Eif4e1b*‐cKO‐derived oocytes. The two neighboring bands may represent the isoforms of eIF4E1B proteins. Additionally, immunofluorescence (IF) analysis indicated that eIF4E1B was barely detectable in *Eif4e1b*‐cKO GV oocytes (Figure 2B). Both of the results showed that no expression of the *Eif4e1b* transcript was detected in *Eif4e1b*‐cKO‐derived oocytes. These findings suggest an efficient elimination of eIF4E1B.

*Eif4e1b*‐cKO female mice were found to be completely infertile in fertility testing, different from WT female mice, which gave birth to approximately six litters per mouse, with an average of about six mice per litter, in the period of 8‐month breeding (Figure 2E). As a result, the oocyte eIF4E1B is essential for female fertility in mice.

To explore how the loss of *Eif4e1b* from oocytes impedes mouse fertility, morphologies and follicle quantification of postnatal day 14, 21, and 35 (PD14, PD21, and PD35 respectively) ovaries from *Eif4e1b*‐cKO mice and their *Eif4e1b*‐WT littermates were all analyzed. We found no apparent morphological difference in the PD14 ovaries of *Eif4e1b*‐cKO female mice. The ovaries of both genotypes had comparable numbers of primordial follicles and follicles surrounded by 2–3 layers of granulosa cells (Figure 1F,G, a magnified image of the respective follicle). At PD21, early secondary follicles (2 layers of granulosa cells) were fewer, and antral follicles were not observed from the growing follicle pool. Besides, *Eif4e1b*‐cKO prepubertal mice had more late secondary follicles (≥3 layers granulosa cells) (Figure 2H,I, a magnified image of the respective follicle). Most of the activated follicles in *Eif4e1b*‐cKO mice arrested at the stage of secondary follicles surrounded by 3–4 layers of granulosa cells, thereby indicating the defective transition from secondary to antral follicles in eIF4E1B‐deficient mice. At PD35 after eCG‐primed 46 h, however, the *Eif4e1b*‐cKO ovaries appeared larger (Figure 2J) with more activated follicles (Figure 2K), including primary, secondary, and antral follicles. The number of follicles at the growing phase was increased, which indicated that follicle recruitment was beyond control in *Eif4e1b*‐cKO mice.

### 2.3 eIF4E1B‐Deficient Embryos are Arrested at the Two‐Cell Stage During OET

**Figure 3:**
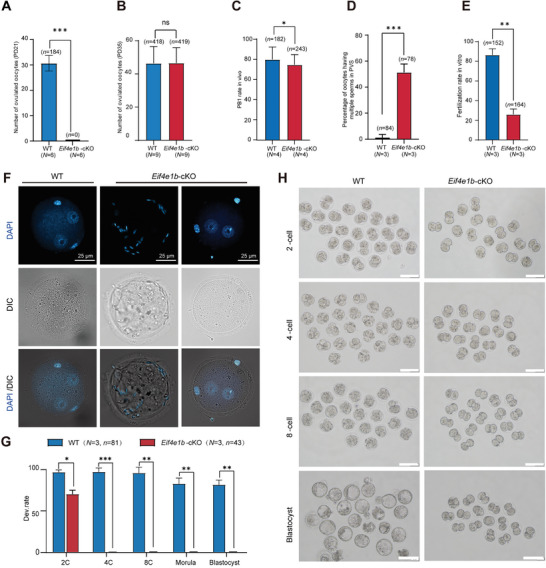
eIF4E1B maternal deficient embryos are arrested at the two‐cell stage during OET. The number of oocytes ovulated by A) PD21 and B) PD35 WT *Eif4e1b*‐cKO females after superovulation. C) Rate of PB1 extrusion of oocytes ovulated by PD35 WT and *Eif4e1b*‐cKO females. D) Percentage of oocytes having multiple sperms in perivitelline space (PVS) rate and E) Fertilization rate of zygotes derived from PD35 WT and *Eif4e1b*‐cKO females. F) Accumulation of sperm in the PVS of the eggs recovered from WT and *Eif4e1b‐*cKO females when fertilized with normal sperms in vitro. Sperm were stained with DAPI. G) Rate of two‐cell and blastocyst formed by ovulated WT and *Eif4e1b*‐cKO females oocytes after IVF. H) Representative images of preimplantation embryos that are derived from WT and *Eif4e1b*‐cKO females at the time when WT embryos are at the corresponding stages. Scale bar, 100 µm. *n*, number of cells; *N*, number of the experiment performed.

In order to understand the potential involvement within oocyte maturation of eIF4E1B, we compared the number of ovulated oocytes first. *Eif4e1b*‐cKO females ovulated an average of 0 and 47 oocytes at PD21 and PD35 respectively, compared with an average of 31 and 46 ovulated oocytes in WT females (**Figure** **3**A,B). The significant difference in the number of MII‐stage oocytes derived from PD21 *Eif4e1b*‐cKO female mice was consistent with its defective follicle development at PD21 with no antral follicle observed.

During the process of oocyte meiotic maturation, the GVBD rate in *Eif4e1b*‐cKO oocytes at PD35 was lower, whereas not statistically significant (Figure S2A, Supporting Information). The polar body‐1 (PB1) extrusion rate was slightly lower than normal, while the spindle assembly and chromosome arrangement had no significant defects in *Eif4e1b*‐cKO oocytes (Figure 3C; Figure S2B,C, Supporting Information).

To further investigate the oocyte developmental competence of *Eif4e1b*‐cKO oocytes at PD35, the process of fertilization was monitored by in vitro fertilization (IVF). During this process, it can be found the accumulation of a lot of sperms in the perivitelline space of the eggs (Figure 3D,F). Also, these defects were shown by live imaging (Movie S1–2, Supporting Information). Although multiple spermatozoa were detected in the PVS, a significant difference in polyspermy in the zygote of *Eif4e1b*‐cKO‐derived oocytes was not observed (Figure S2D–F, Supporting Information). It was found that only ≈25.94% of the *Eif4e1b*‐cKO ovulated oocytes were successfully fertilized and 70.02% of them developed to the two‐cell stage after fertilization with normal sperm (Figure 3E,G). No fertilized *Eif4e1b*‐cKO oocytes developed beyond the two‐cell stage in further culture (Figure 3H). In sum, the *Eif4e1b*‐cKO oocyte had a severely impaired developmental competence, suggesting a maternal origin of the developmental phenotype.

### 2.4 eIF4E1B Binds mRNA Targets Required for OET

**Figure 4:**
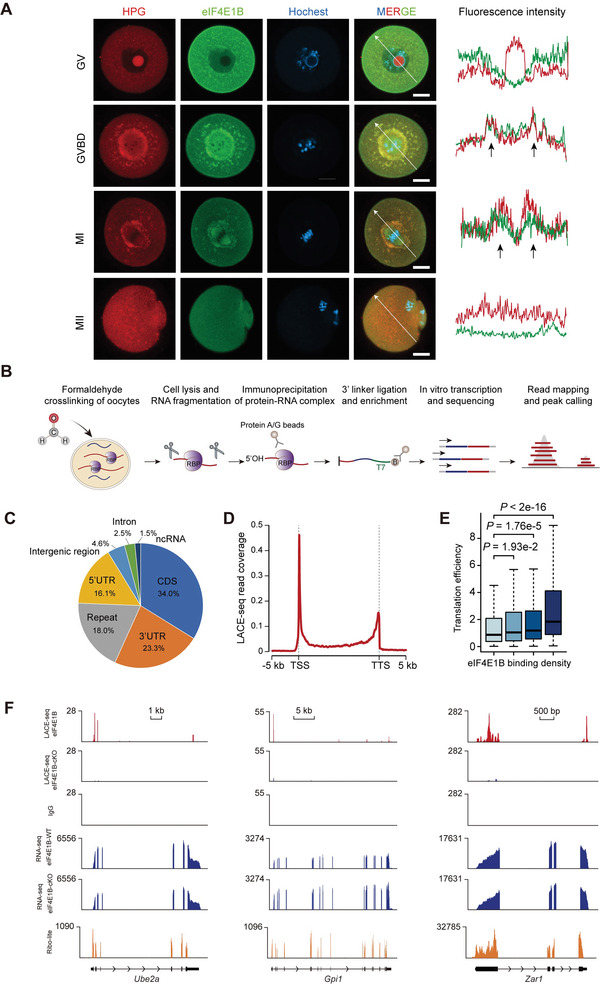
The binding landscape of eIF4E1B. A) Oocytes overexpressed with EGFP‐eIF4E1B cRNA in various stages were incubated in the medium for 30 min with HPG. HPG (red); eIF4E1B‐EGFP (green); DAPI (blue). For the histogram in the right panel of GVBD and MI, it is shown that HPG (red) and eIF4E1B (green) intensity are depicted along the white line. Both the CTA and PTA are indicated by the arrowhead. Scale bar, 25 µm B) Schematic of the LACE‐seq method to profile eIF4E1B binding sites within oocytes. C) Genomic distribution of eIF4E1B binding sites that are revealed by LACE‐seq. D) Meta profile which presents eIF4E1B LACE‐seq reads distribution around the gene body. TSS: transcription start site; TTS: transcription termination site. E) Boxplots that present the correlation between the eIF4E1B binding density and TE detected by Ribo‐lite in GV oocytes. eIF4E1B binding RNAs involved four equal categories based on LACE‐seq binding density (from low to high, Q1 to Q4, and an average of 447 RNAs for every quarter). P values were determined by the two‐tailed Wilcoxon test. F) The UCSC browser views of eIF4E1B LACE‐seq reads for *Ube2a*, *Gpi1*, and *Zar1* within mouse GV oocytes. Red track, eIF4E1B LACE‐seq; eIF4E1B‐cKO LACE‐seq and IgG LACE‐seq track are shown below; Blue track, the peaks information of RNA‐Seq in WT and *Eif4e1b*‐cKO GV oocytes; Orange track below, Ribo‐lite.

The initiation of preimplantation development and oocyte maturation depends on the post‐transcription of mRNAs transcribed in growing oocytes.^[^ ^1^ ^]^ It has been shown that oocytes translate de novo proteins located in chromosome and perispindular translational areas (CTA and PTA) during maturation.^[^ ^20^ ^]^ Emerging proteosynthesis and the spatio‐temporal distribution of eIF4E1B during oocyte maturation were analyzed by overexpressing fluorescent protein‐tagged eIF4E1B and culturing fully grown oocytes (FGOs) in a medium with homopropargylglycine (HPG) for a short period of cultivation. Interestingly, eIF4E1B was distributed with a similar pattern of nascent proteosynthesis after meiotic resumption (**Figure** **4**A). The results indicated that the localization of eIF4E1B correlated with de novo proteins in CTA and/or PTA where translation may have increased.

As a classic RNA‐binding protein, a low‐input CLIP‐seq technology, named LACE‐seq we recently developed was performed to identify mRNAs loaded onto eIF4E1B by using eIF4E1B immunoprecipitated (Iped) mRNAs from oocytes in mouse GV stage (Figure 4B). In order to exclude the detected false positive signals, the eIF4E1B LACE‐seq in *Eif4e1b*‐cKO oocytes and  (IgG) LACE‐seq using the control IgG rabbit serum antibody in WT oocytes were carried out simultaneously. We found that eIF4E1B binding signals were significantly reduced in *Eif4e1b*‐cKO oocytes (Figure S3A, Supporting Information). The peaks identified in the WT group with fivefold less enrichment than the IgG group and *Eif4e1b*‐cKO group were removed as false positive signals. The remaining peaks were considered as eIF4E1B binding peaks (Figure S3B, Supporting Information). Two eIF4E1B LACE‐seq libraries were highly reproducible (*R* = 0.92, Figure S3C, Supporting Information), and the two biological replicates were combined for downstream analysis. Of the 4227 eIF4E1B binding sites, ≈34.0% were located in the coding sequence (CDS), while 39.4% were derived from UTR. 18.0% of eIF4E1B binding sites were located in repetitive elements (Figure 4C). In the meta profile, it can be found that eIF4E1B was preferentially bound to the transcription start site (TSS) and transcription termination site (TTS) of genes (Figure 4D), therefore indicating that eIF4E1B interacts with the 5′ UTR and 3′UTR of the mRNAs. These binding rules coincide with the role of eIF4E1B in translation initiation. To further investigate the role of eIF4E1B on translation regulation, we downloaded the ribosome profiling data (Ribo‐lite)^[^ ^30^ ^]^ and compared the translation efficiency (TE) between eIF4E1B‐bound targets and no‐bound targets. We found that eIF4E1B‐bound targets had higher TE than no‐bound targets, and eIF4E1B targets with higher binding density had stronger TE, thereby indicating that the eIF4E1B binding identity is coupled with TE. (Figure 4E; Figure S3D,E, Supporting Information).

In the network of GO analyses, it is demonstrated that eIF4E1B targets (*n* = 1921) were enriched for the terms associated with the regulation of translation, cell division, chromatin organization, and ubiquitin‐dependent protein catabolic process (Figure S3F, Supporting Information). Taking into account the severe defects of fertilization and 2‐cell block phenotypes observed in *Eif4e1b*‐cKO oocytes, we noticed *Ube2a, Gpi1*, and *Zar1* which had reported functions in gamete fusion and early embryo development.^[^ ^34^, ^35^, ^36^, ^37^, ^38^ ^]^ The eIF4E1B LACE‐seq signals peaked around the 5′ UTR and 3′ UTR of these genes, which coincides with eIF4E1B binding rules (Figure 4F). Importantly, there was no significant reduction of transcript levels after the depletion of *Eif4e1b*. Taken together, these results indicate that *Eif4e1b* mainly functions in the translation initiation of targeted mRNAs that are required for various processes during OET.

### 2.5 eIF4E1B Selectively Activates Translation Through 5′UTR via CG‐Rich Motif

**Figure 5:**
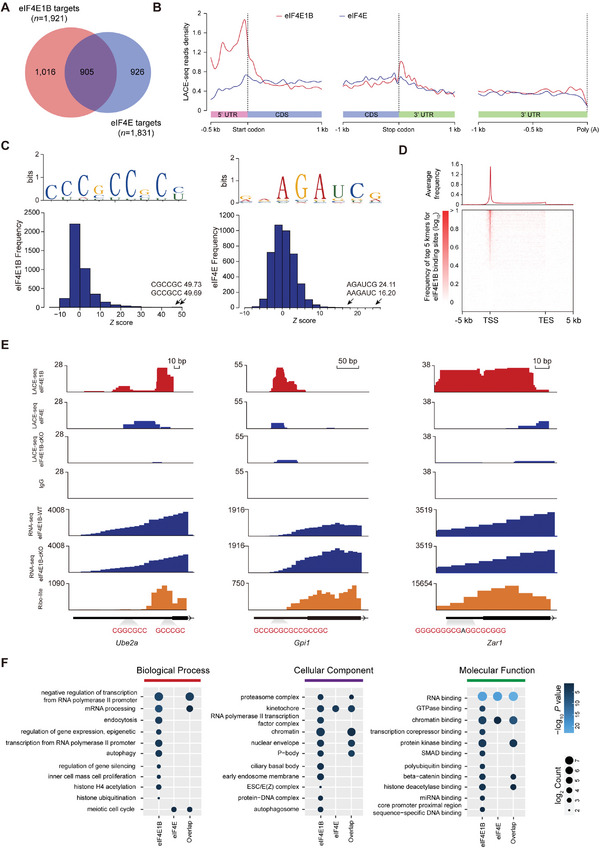
eIF4E1B selectively activates translation through 5′UTR via CG‐rich motif. A) Venn diagram which presents the direct overlap from eIF4E1B (red) to eIF4E (blue) targets. eIF4E1B‐specific targets, *n* = 1016; eIF4E‐specific targets, *n* = 926. B) Meta profile showing the eIF4E1B (red) and eIF4E (blue) binding signals around the start codons (left), stop codons (middle), and poly(A) sites (right). C) Histogram showing overrepresented eIF4E1B (left) and eIF4E (right) binding motifs that are identified by LACE‐seq. Z scores of the top‐two hexamers were indicated. In the upper, the binding consensus calculated from the top 20 enriched hexamers is shown. D) Metaplot and heatmap showing the distribution of top 5 hexamers identified by eIF4E1B binding sites around gene body. E) The UCSC browser views of LACE‐seq reads and Ribo‐seq signal (RPF) for *Ube2a*, *Gpi1*, and *Zar1* within mouse GV oocytes. Red track, eIF4E1B LACE‐seq; eIF4E1B‐cKO LACE‐seq and IgG LACE‐seq track are shown below; Blue track, the peak information of RNA‐Seq in WT and *Eif4e1b*‐cKO GV oocytes; Orange track below, Ribo‐lite. A magnified view of eIF4E1B binding motifs around 5′ UTR is shown at the bottom. F) Dotplot visualization for enriched GO terms of eIF4E1B‐specific, eIF4E‐specific, and their overlapped targets in mouse GV oocytes. The dot color serves as the *p*‐value for every enriched GO term, and the size refers to the number of genes that are enriched within every GO term.

To further understand the mechanism of the specificity of eIF4E1B binding patterns for regulating the translation initiation of key genes, eIF4E, a classical translation activation factor that widely binds the majority of capped mRNAs in the cytoplasm, was selected for LACE‐seq analysis.^[^ ^21^, ^39^, ^40^ ^]^ Two reproducible LACE‐seq libraries were combined for downstream analysis (*R* = 0.86, Figure S3G, Supporting Information). Similar to eIF4E1B, ≈53.9% of the eIF4E binding sites were located in the CDS, while only 24.5% were derived from UTR. 11.5% were located in repetitive elements (Figure S3H, Supporting Information). It was found that 47.1% of the eIF4E1B targets identified by LACE‐seq were also the eIF4E targets (**Figure** **5**A; Table S1, Supporting Information), suggesting that there is a functional overlap. Unexpectedly, the meta‐analysis revealed that eIF4E1B had a more predominant binding strength around the start codon and 5′ UTR than eIF4E (Figure 5B), indicating a biased binding affinity of eIF4E1B to the 5′ UTR of target mRNAs. To investigate the consensus binding motifs for eIF4E1B and eIF4E, the overrepresented hexamers were identified at the binding sites, and the most abundant motif enriched at the eIF4E1B binding sites was found to closely match GC‐rich hexamers (Figure 5C). Of the 4227 eIF4E1B binding sites (with an average length of 51.5 nt), at least one GC‐rich hexamer was contained at 43.6% of the peaks, and at least one top‐20 motif was contained at 46.7% of the peaks (Figure 5C). In contrast, the most enriched hexamer at binding sites for eIF4E proteins was AGAUCG (Figure 5C). Furthermore, the GC‐rich hexamers were significantly enriched around TSS (Figure 5D), contributing to the eIF4E1B binding preference around 5′ UTR. *Ube2a, Gpi1, and Zar1* with GC‐rich motifs were observed to have stronger eIF4E1B LACE‐seq signals around 5′ UTRs compared to eIF4E, where the ribosome‐protected fragment (RPF) was detected by Ribo‐lite (Figure 5E). Given that part of the RNAs were co‐bound by eIF4E1B and eIF4E, we sought to explore their specific biological functions. By comparing the GO terms among eIF4E1B‐ (*n* = 1016), eIF4E‐specific targets (*n* = 926) and their overlapped targets (*n* = 905), we found eIF4E1B‐specific targets illustrate a stronger enrichment of GO terms, including the biological process like regulation of gene expression (epigenetic), transcription from RNA polymerase II promoter, and histone H4 acetylation; and cell components like P body and ciliary basal body as well as the molecular function in GTPase and polyubiquitin binding (Figure 5F; Table S2, Supporting Information).

Together, these results demonstrate that eIF4E1B has specifical binding preference via CG‐rich motif in 5′UTR and play a role in the selective translation of key genes involved in OET.

### 2.6 Proteomics Analysis of eIF4E1B‐Mediated Selective Translation

**Figure 6:**
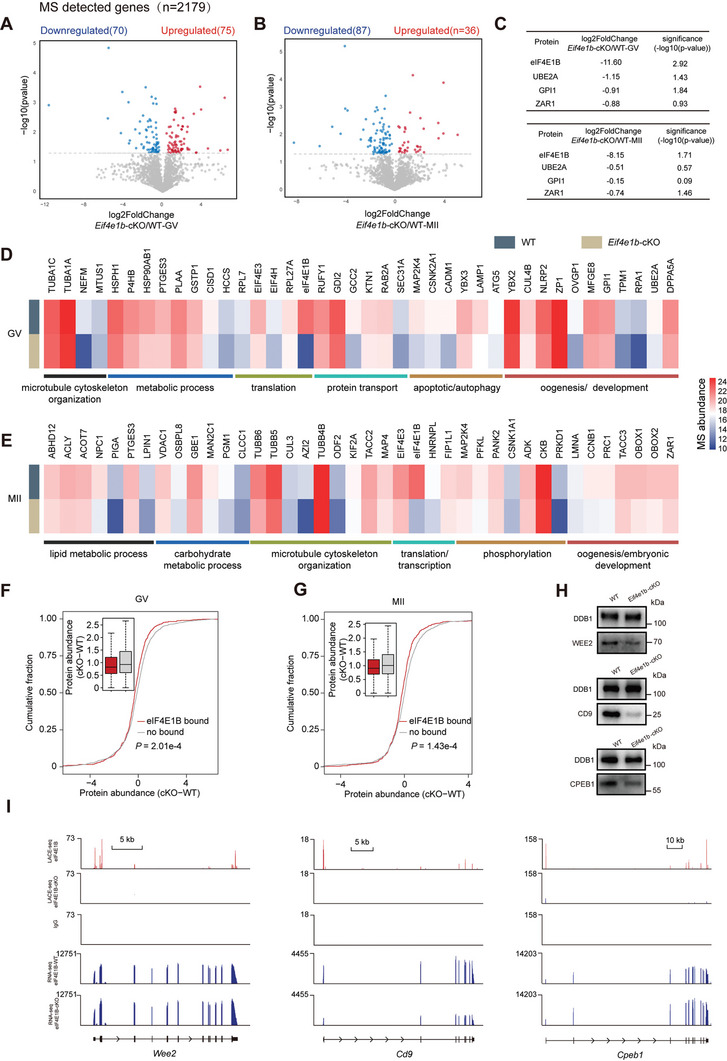
Proteomics analysis of eIF4E1B‐mediated selective translation. A,B) The Thermo Fisher Orbitrap Eclipse Tribrid mass detected a total of 2179 protein‐related genes that were applied for exploration. Significantly upregulated (red) and downregulated (blue) proteins within *Eif4e1b*‐cKO GV and MII oocytes (*p* < 0.05). C) Fold change and significance of *Ube2a*, *Gpi1*, and *Zar1* protein levels disclosed by mass spectrometry within *Eif4e1b*‐cKO GV and MII oocytes. D,E) Heatmaps that present differences from WT to *Eif4e1b*‐cKO oocytes within the cohort expression of downregulated proteins of a variety of processes. F,G) *Eif4e1b* ‐bound genes presented stronger downregulation at the protein levels compared to the unbound genes within GV and MII *Eif4e1b* ‐cKO oocytes. The *p*‐values in F and G were determined by the two‐tailed Wilcoxon test. MS data from every group within the findings includes three independent experiments. (H) Western blotting results present endogenous protein levels of WEE2, and CD9 within MII oocytes and CPEB1 within GV oocytes. DDB1 was blotted as the loading control. The experiment went through three independent repetitions and reached similar findings. I) The UCSC browser views of LACE‐seq reads for *Wee2*, *Cd9*, and *Cpeb1* in mouse GV oocytes. Red track, eIF4E1B LACE‐seq; eIF4E1B‐cKO LACE‐seq and IgG LACE‐seq track are shown below; Blue track, the peaks information of RNA‐Seq in WT and *Eif4e1b*‐ cKO GV oocytes.

To verify that *Eif4e1b*‐targeted mRNA changed on the translation level, the protein‐expression profile of *Eif4e1b*‐cKO oocytes was compared to that of WT GV and MII oocytes by liquid chromatography‐mass spectrometry (LC‐MS) through using a Thermo Scientific Orbitrap Eclipse Tribrid mass spectrometer. Biological replicates indicated a strong correlation within the detected proteins (Figure S4, Supporting Information), and a total of 2179 proteins were detected, with 145 and 123 differentially expressed by *Eif4e1b*‐cKO GV and MII oocytes (*p* < 0.05) (**Figure** **6**A,B). In Tables S3 and S4 (Supporting Information), these differentially expressed proteins after *Eif4e1b* deletion in oocytes at the GV and MII stage are shown. The eIF4E1B serves as one of the most significantly down‐regulated proteins. In consistent with the previous targets detected by eIF4E1B LACE‐seq, the expression of UBE2A, GPI1 and ZAR1 were downregulated at different degrees in GV and MII oocytes (Figure 6C).

Efforts were then made to understand how these changed proteins affect oocyte maturation and development competence, especially the down‐regulated factors that may be directly activated by eIF4E1B. Quantitative analysis of the proteomic data revealed that 70 proteins were down‐regulated and 75 proteins were up‐regulated in *Eif4e1b*‐cKO GV oocytes, GO analysis indicated that the decreased proteins were involved in the process including microtubule cytoskeleton organization, metabolism process, and translation (Figure 6D).

By comparing the detected proteins of MII oocytes derived from WT and *Eif4e1b*‐cKO female mice, it was found that 87 proteins were down‐regulated and 36 proteins were up‐regulated in *Eif4e1b*‐cKO MII oocytes. Furthermore, GO analysis of the down‐regulated proteins revealed that the terms related to lipid metabolic process and carbohydrate metabolic processes were enriched (Figure 6E). Accordingly, insufficient translation of proteins at different stages is essential for a variety of processes in *Eif4e1b*‐cKO oocytes, leading to eIF4E1B‐deficient embryos do not develop beyond the two‐cell stage.

To determine whether eIF4E1B‐deletion‐related proteomics changes were directly regulated by its role in translational instead of transcriptional regulation, we conducted the single‐cell RNA‐seq data in mouse WT and *Eif4e1b* ‐cKO oocytes at the GV and MII stages. In *Eif4e1b*‐cKO oocytes at the GV stage, it can be found that 611 genes were up‐regulated, and 791 genes were down‐regulated (Figure S5A, Supporting Information); and at the MII stage, it can be seen that 409 genes were up‐regulated, and 465 genes were down‐regulated (Figure S5B, Supporting Information). Among the significantly changed proteins detected, we identified 9 and 3 genes were differentially expressed on mRNA levels in the GV and MII oocytes from *Eif4e1b*‐cKO females (Figure S5C, Supporting Information). Among the down‐regulation of 70 and 87 proteins at the GV and MII stages, only 2 and 3 genes were downregulated on mRNA level respectively (Figure S5D, Supporting Information). Therefore, the changes in proteomics are seen to correlate poorly with their mRNAs.

To further verify the relationship between eIF4E1B‐deletion‐related proteomics and *Eif4e1b‐*targeted mRNA, 1921 direct targets of *Eif4e1b* identified by LACE‐seq were compared with the proteomics data. Among the 70 and 87 down‐regulated proteins in *Eif4e1b*‐cKO GV and MII oocytes, 31.4% (GV) and 36.8% (MII) were direct targets of eIF4E1B identified by LACE‐seq (Figure S5E,F, Supporting Information). As for the 75 and 36 up‐regulated proteins in *Eif4e1b*‐cKO GV and MII oocytes, 14.7% (GV) and 13.9% (MII) were direct targets of eIF4E1B identified by LACE‐seq (Figure S5G,H, Supporting Information), thereby indicating their possibility to be direct targets of *Eif4e1b*‐translation activation complexes. Interestingly, *Eif4e1b*‐bound genes showed higher downregulation at the protein level than the unbound group both in GV (*P* = 2.01× 10^−4^; Figure 6F). and MII oocytes (*P* = 1.43× 10^−4^; Figure 6G). In addition, our proteomics analysis further confirmed the selective translation of the targets by eIF4E1B.

Proteins are not expandable, thereby making it difficult to identify the potentially larger groups of proteins regulated by eIF4E1B by only using MS in these rare oocytes. Next, some vital maternal factors, such as *Wee2*, *Cd9*, and *Cpeb1*, were picked, and eIF4E1B LACE‐seq signals peaked around the 5′ UTR and 3′ UTR (Figure 6I).^[^ ^41^, ^42^, ^43^ ^]^ Unexpectedly, it can be seen that WEE2, CD9, and CPEB1 showed consistent downregulation as revealed by western blotting (Figure 6H), even though none of them passed the stringent cut‐off criteria used within our proteomics analysis. These results indicated that the proteomics analysis combined with the eIF4E1B specific binding genes are likely to give a wider insight to understand the potential protein network regulated by eIF4E1B during OET.

### 2.7 eIF4E1B Interacts with 3′UTR‐Associated RBPs to Activate Translation

**Figure 7:**
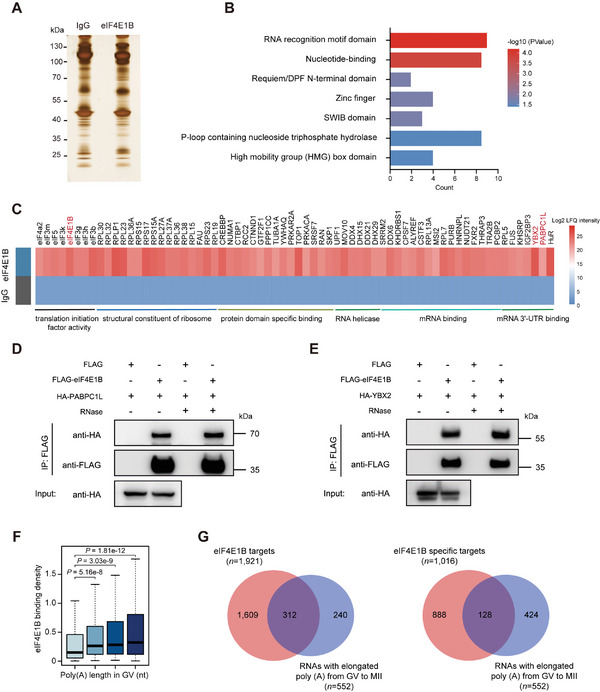
eIF4E1B interacts with 3′UTR‐associated RBPs to activate translation. A) Silver‐stained gel of bound samples that are obtained from mouse ovaries lysates were immunoprecipitated using IgG and ≈35 kDa eIF4E1B antibody, bound proteins eluted and analyzed by SDS‐PAGE. B) Interpro domain analysis of target proteins. C) Heatmap representing proteins present with different abundance in anti‐eIF4E1B Iped complexes within lysis that is prepared from mouse ovaries compared with the IgG Iped negative control. The heatmap was produced through the Multi Experiment Viewer software, in which the lowest abundant proteins are in blue, the intermediate abundant proteins are in white and the highest abundant proteins are in red. D,E) 293T cells in total were co‐transfected with HA‐PABPC1L or HA‐YBX2 expression plasmids for 48 h. RNase A was used to eliminate the impact of RNAs. Target proteins were immunoprecipitated using anti‐FLAG beads and then subjected to western blotting with FLAG and HA antibodies. Input cell lysates were immunoblotted with an anti‐HA antibody to determine the expression of PABPC1L and YBX2. F) Boxplots showing that RNAs with longer poly(A) tail indicated stronger eIF4E1B binding density detected by LACE‐seq in GV oocytes. eIF4E binding RNAs involved four equal categories on the basis of the poly(A) tail length detected by PAIso‐seq (from low to high, Q1 to Q4, and an average of 449 RNAs per quarter). The two‐tailed Wilcoxon test decided *p‐*values. G) Venn diagrams that show the relationship of the eIF4E1B targets (or eIF4E1B‐specific targets) and the polyadenylated genes during the process of maturation identified by PAIso‐seq in GV and MII oocytes.

To further explore the mechanism, a potential cofactor(s) with the eIF4E1B‐ mediated machinery was identified to disclose the target mRNA translational activation. For this purpose, coIP was used for proteomic analysis in anti‐eIF4E1B and IgG control Iped complexes from ovarian lysates and 2500 GV oocytes (**Figure** **7**A; Figure S6A, Supporting Information). A total of 787 proteins were detected in anti‐eIF4E1B Iped complexes from ovarian lysates, with 308 proteins being specificity pulled down (Figure S6B, Supporting Information). However, in the Iped complex from GV oocytes, 44 out of 170 detected proteins were specificity pulled down (Figure S6C, Supporting Information). The InterPro domain analysis revealed that eIF4E1B‐interacting proteins were enriched in the RNA recognition motif (RRM) (Figure 7B; Table S5, Supporting Information).

GO analysis of the candidate proteins identified in the proteomic screen revealed that translation initiation factors including eIF4a2, eIF3b, eIF3g, eIF3k, eIF3m, and eIF5 were enriched in the molecular function of “translation initiation factor activity”. In addition, other proteins such as RPL isoforms play a role in the structure of the ribosome, with MOV10, DDX4, and DHX29 as RNA helicases. Notably, the majority of mRNA binding proteins such as SRSF7, CPSF7, HNRNPL, and mRNA 3′UTR binding proteins such as YBX2, IGF2BP2/3, PABPC1L, and HuR/HuB were identified as co‐factors with eIF4E1B (Figure 7C; Figure S6E, Supporting Information). This indicates the formation of large RNP complexes in collaboration with eIF4E1B involved in translation initiation.

To further confirm the interaction between eIF4E1B and target proteins, YBX2, IGF2BP2, and PABPC1L were chosen for further verification by overexpressing the FLAG‐tagged eIF4E1B and HA‐tagged YBX2, IGF2BP2 or PABPC1L in 293T cells, which are mRNA 3′UTR binding proteins and vital proteins for female fertility(Figure S6D, Supporting Information).^[^ ^44^, ^45^, ^46^ ^]^ It was found that the FLAG‐tagged eIF4E1B could pull down the HA‐tagged YBX2, and PABPC1L in vitro in the presence or absence of RNase treatment (Figure 7D,E). This suggests that eIF4E1B has interaction with these 3′UTR‐associated RBPs independent of RNA.

In the process of translation, PABPs mediate the formation of the “closed‐loop complex”, where the ends of mRNA connect the interaction between PABP and the cap‐bound translation initiation complex.^[^ ^18^ ^]^ Interestingly, in the three PABP isoforms, only *Pabpc1l* mRNA is expressed from oocytes to two‐cell embryos^[^ ^46^ ^]^ (Figure S6F, Supporting Information). A report has also observed that PABPC1L achieves translation activation by elongation of maternally‐stored mRNAs upon stimulation of oocyte maturation, which is important for female fertility in mice.^[^ ^46^ ^]^ These findings point to the requirement for an interaction between PABPC1L and eIF4E1B in activating maternal mRNA translation through poly (A) tail elongation. To further validate this model in oocytes, the poly (A) inclusive RNA isoform sequencing (PAIso‐seq) data in GV and MII oocytes were downloaded to identify how to regulate poly (A) tails in the process of meiosis.^[^ ^47^ ^]^ By comparing genes with poly (A) tails, it was found that the longer the length of poly (A) tails with RNAs, the stronger the eIF4E1B binding density detected by LACE‐seq in GV oocytes (Figure 7F). Of the 552 polyadenylationized RNAs from GV to MII oocytes, 312 (56.5%) genes were targeted by eIF4E1B, with 128 (23.2%) genes as specific targets (Figure 7G; Table S6, Supporting Information). Notably, the majority of mRNAs polyadenylationized during the GV‐to‐MII transition may be direct targets of *Eif4e1b* translation activation complexes.

To sum up, these findings indicate that eIF4E1B may function together with 3′UTR‐associated RBPs to activate translation.

## 3 Discussion

**Figure 8:**
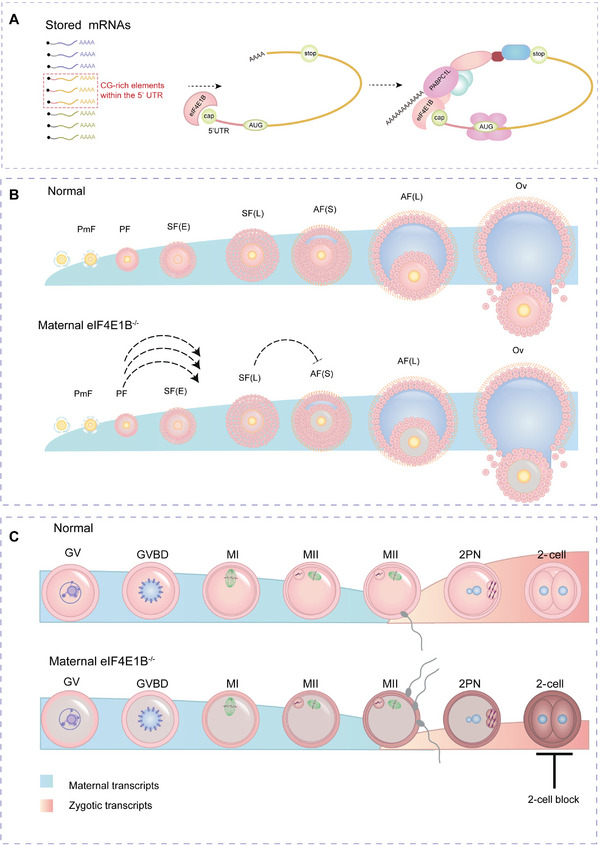
Selective translation of maternal mRNA by eIF4E1B controls oocyte‐to‐embryo transition. A) A complicated mixed population of derepressed mRNAs shown in oocytes of various stages is selectively recruited for translation initiation by the individual eIF4 isoforms. The eIF4E1B‐based selective translation via biased binding of the CG‐rich element within 5′UTR of targets mRNA and interacts with the 3′UTR‐associated RBPs to form the “closed‐loop complex” in new protein synthesis which temporally occurs during the process of OET. B,C) The temporal and spatial translation of maternal transcripts serves as a significant component within the cytoplasmic maturation process of oocytes. A distinct subset of mRNAs is recruited to the cap‐dependent translation initiation complex through the eIF4E1B. The maternal deletion of eIF4E1B results in the impaired translation of proteins in the process of oogenesis, fertilization, and early embryo development and ultimately 2‐cell development arrest. Abbreviations: PmF, Primordial follicles; PF, Primary follicles; SF(E), Early secondary follicles; SF(L), Late secondary follicles; AF(S), Small antral follicles; AF(L), Large antral follicles; GV, germinal vesicle oocyte; GVBD, germinal vesicle break down oocyte; MI, metaphase I oocyte; and MII, metaphase II‐arrested oocyte. In addition, the blue color below indicates the maternal transcripts, and the red color below shows the zygotic transcripts.

Previous studies have shown specific translational control of several germline mRNAs by cytoplasmic polyadenylation on the properties of 3′UTR,^[^ ^13^, ^15^, ^36^, ^48^ ^]^ but it remains an open question that how a large number of depressed mRNAs are selectively activated in mammalian oocytes. Herein, the deletion of a mammalian eukaryotic translation initiation factor eIF4E1B was reported, and the physiological importance of the eIF4E1B‐ mediated selective translation machinery involved in regulating translation during OET was also identified (**Figure** **8**). On the whole, these findings indicate as follows: i) eIF4E1B expressed in oocytes from primary follicles to antral follicles and sustained to 2‐cell embryos; ii) eIF4E1B‐deficient oocytes had multiple defects concerning oogenesis and embryonic developmental competence during OET; iii) eIF4E1B can selectively target the mRNAs characterized by CG‐rich elements in 5′UTR and stimulate the translation efficiency; iv) eIF4E1B functions together with 3′UTR‐associated RBPs involved in stabilizing mRNA stability and promoting translation activation.

The eIF4E was originally considered as a single protein of a single 25‐kDa polypeptide obtained from a mammalian origin.^[^ ^21^ ^]^ When the wheat germ was unexpectedly found to contain two versions of eIF4E, it was later reported that almost all eukaryotes expressed multiple eIF4E family members in germ cells. In *Xenopus* and zebrafish, eIF4E1b is an isoform of eIF4Es, whose expression is confined to oocytes, eggs, and early embryos in mice.^[^ ^21^, ^22^, ^23^, ^24^, ^25^ ^]^ By comparison, eIF4E1B can distribute a similar expression pattern during OET in mice, which is a specific subcellular distribution characterized by colocalizing with the signals of de novo protein synthesis during oocyte maturation. In the germ line of *C.elegans*, eIF4E (IFE‐1) is a unique germline isoform of eIF4E needed to efficiently translate the maternal mRNAs. The deletion of IFE‐1 by RNAi modestly reduces the oocyte production and fertility of wild‐type spermatozoa.^[^ ^23^ ^]^ Herein, targeted disruption of the *Eif4e1b* gene in mice eventually distributed defects in oogenesis and development arrest at the two‐cell stage. In the findings, eIF4E1B is suggested to be an important maternal factor with a conserved mechanism in reproduction during the process of evolution.

Through defined binding domains, the RBP interacts with RNA to regulate the RNA fate, such as translation activation/repression, deadenylation, poly (A) tail elongation, stability, and localization.^[^ ^6^ ^]^ Previous studies have documented many maternal RBPs in mice, including CPEB1, DAZL, ZAR1, PABPC1L, YTHDF2, and IGF2BP2,^[^ ^11^, ^16^, ^35^, ^44^, ^48^, ^49^ ^]^ having essential functions at various aspects in mRNA processing, translation, and degradation. However, in the low‐abundance oocytes, it is difficult for current methods to identify the in vivo protein‐RNA interaction sites. As a result, the functional mechanisms are poorly understood. The mRNA loaded on the eIF4E1B‐specific machinery represents the mRNA metabolism, especially for translational repression or activation during OET. Indeed, by using LACE‐seq, a group of 1921 mRNAs were identified as direct targets of eIF4E1B in GV oocytes, and LC‐MS/MS analysis further demonstrated that some genes were regulated at the protein level. These findings provide a genome‐wide representation of transcripts regulated by eIF4E1B, revealing a widespread involvement of its selectively translational regulation in oocytes.

Although eIF4E stimulates the translation of capped mRNAs, mRNAs depend differently on eIF4E.^[^ ^23^ ^]^ It has been reported that eIF4E is distributed evenly and also present in the vicinity of chromosomes after GVBD during oocyte maturation.^[^ ^50^ ^]^ Through a comparison of the eIF4E1B targets with eIF4E targets, it was found a group of genes overlapped with the genes targeted to eIF4E1B. GO analysis revealed the overlapped function between eIF4E1B and eIF4E targeted genes including the process of the meiotic cell cycle (Figure 5F). The weak phenotype of meiotic in *Eif4e1b*‐cKO oocytes such as slightly lower PB1 extrusion rate, normal spindle assembly, and chromosome arrangement suggested eIF4E may compensate for the loss of eIF4E1B in the process of meiotic.

Moreover, it was found that a unique group of maternally transcribed genes targeted to eIF4E1B, demonstrated the distinct biochemical factors in OET that are irreplaceable by eIF4E. The potential element was defined within the 5′UTR of eIF4E1B‐specific targets, suggesting a significant binding preference around start codons and 5′UTR of targets mRNA. This indicates that a CG‐rich motif in the region may have greater requirements for the eIF4E1B‐based translation machinery. Because RNA molecules can fold into complex shapes and provide an additional layer of gene expression control beyond the sequence. Hence, novel approaches were proposed, which can be used to enhance an understanding of the functional and contributing features between CPE, AAUAAA within 3′UTR, and CG‐rich elements within 5′UTR. Future studies may focus on the secondary structure necessary for effective cytoplasmic RNA translation activation.^[^ ^17^, ^19^ ^]^

The 5′ cap and 3′ poly (A) tail were discovered in all RNA polymerase II transcripts. Correspondingly, the two core factors of translation initiation refer to the translation initiation factor eIF4Es bound to the 5′ cap and the PABPs bound to the poly (A) tail. Together with the eIF4G, eIF4Es and PABPs mediate the so‐called mRNA closed‐loop structure. Using LACE‐seq, it was found that eIF4E1B targeted the 5′ and 3′ ends of some transcripts, providing physical evidence for the reported closed‐loop model. The biochemical data in vitro illustrated the interaction between eIF4E1B and mRNA 3′‐UTR binding proteins such as YBX2 and PABPC1L. As multiple eIF4E family members in germ cells, PABPs also have distinct expression patterns.^[^ ^51^ ^]^ These results suggest that it is possible to assume a potential oocyte‐specific closed‐loop‐model: eIF4E1B‐eIF4G‐PABPC1L, which can be used to control various target genes to promote the stability and translation efficiency of multiple developmental events during OET.

In conclusion, the above findings uncover an oocyte‐specific role of eIF4E1B in selecting and regulating the translation initiation of individual mRNAs from a competing pool of mRNAs in mammalian species. The differential proteins and targets identified in this paper may provide candidates for further elucidating vital biological processes during OET. The potential genetic markers are also required to identify the reasons for female infertility characterized by meiotic maturation arrest, fertilization failure, or early embryonic development arrest in clinical practice. Moreover, the mechanism of translation regulation revealed in this paper may give an insight into the potential role of the human ortholog of eIF4E1B.

## 4 Experimental Section

### Laboratory Mice and Fertility Test

All the mice used for experiments were raised and bred under standard conditions at the Department of Laboratory Animals in Central South University (Changsha, P. R. China) whose Institutional Animal Care and Use Committee authorized mouse experimental procedures and protocols (Approval number: 2021sydw0263). *Eif4e1b* ^loxP/loxP^ mice were generated by the Cyagen Biosciences Institute. *Eif4e1b* ^loxP/+^; Zp3‐Cre progenies, which were then used for the creation of oocyte‐specific *Eif4e1b*‐KO mice in growing oocytes. To obtain *Eif4e1b* ^loxP/loxP^; Zp3Cre mice, the *Eif4e1b* ^loxP/+^; Zp3‐Cre males were hybridized with *Eif4e1b* ^loxP/loxP^ females. Then, colonies were conventionally kept in a breeding plan that produced either *Eif4e1b* ^loxP/loxP^ or *Eif4e1b* ^loxP/loxP^; Zp3Cre (*Eif4e1b*‐cKO for short) females that were used for experiments according to the previous strategy.^[^ ^52^ ^]^ All of these mice were raised under the same C57BL/6J genetic background and genotyped by polymerase chain reaction (PCR) by using primers in Table S7 in the Supporting Information. Six‐week WT and *Eif4e1b*‐cKO females were mated with ordinary adult C57BL/6J males to perform the breeding and fertility test of female mice for eight months. The number of pups per litter was recorded at birth, and the mean number of pups per litter was calculated in the late testing period.

### RNA Isolation and Quantitative RT‐PCR

In accordance with the manufacturer's instructions, the Total RNA Extraction Kit (Promega, LS1040) was used to extract total RNA, and RNA was reversely transcribed via the Super Script IV (Invitrogen, Catalog #18090010). Oligo(dT)12‐18 (Invitrogen, Catalog #18418012) was used to guide the reverse transcription. RT‐qPCR was carried out by using an Applied Biosystems 7500 Real‐Time PCR System and Power SYBR Green PCR Master Mix (Applied Biosystems, Life Technologies). A fold change in relative transcription levels was determined by comparing them with endogenous *Gapdh* (internal control). Three replicates of RT‐qPCR experiments were carried out. Table S8 (Supporting Information) presents the primer sequences.

### Histological Analyses

Ovaries from PD14, PD21, PD35 *Eif4e1b*‐cKO, and *Eif4e1b*‐WT mice were embedded in paraffin, and serial sections of 5 µm in thickness were prepared and stained with periodic acid‐Schiff reagent (PAS) and Lillie‐Mayer hematoxylin. With the counting of the total number of per‐stage follicles, follicles at various development phases were calculated in each third section of the whole ovary. For the purpose of avoiding the repetitive calculation of the same follicle, the calculation was carried out on just follicles that involve an oocyte with visible GV. In accordance with the prior mentioned criteria, follicles stages were categorized.^[^ ^52^ ^]^

### Oocyte Isolation and in Vitro Maturation, Fertilization, and Embryo Culture

Cumulus‐oocyte complexes (COCs) were separate from the large antral follicles of equine chorionic gonadotropin (eCG, Beijing Solarbio Science & Technology, Catalog # P9970)‐primed (46 h) 21‐day‐old female mice, and the cumulus cells of these COCs were removed to obtain denuded oocytes (DOs) as described previously.^[^ ^52^ ^]^ DOs got mature in the culture, and then oocyte samples at GV, GVBD, M I, and M II were gathered at the time points of 0, 3, 6, and 14 h during the process of culture for the immunofluorescence (IF) staining of eIF4E1B, microtubules and chromosomes. Ovulated oocytes were obtained from the ampulla of oviducts following a super‐ovulation regimen for mice. Oocytes were collected and cultured in 37 °C pre‐warmed modified eagle medium (MEM) with Earle's salts (Gibco, Catalog # 11095), and added with 75 µg ml^−1^, 50 µg ml^−1^, 0.23 mM, and 3 mg ml^−1^ of penicillin G, streptomycin sulfate, pyruvate, and bovine serum albumin respectively at 37 °C in 5% CO_2_ in compressed air and high humidity. Concerning IVF, normal sperms isolated from C57BL/6J adult males were fertilized with ovulated oocytes in vitro. 24 h after IVF, the formation of two‐cell stage embryos transferred into an M16 culture medium (Sigma Aldrich, Catalog #M7292) for subsequent development was scored. Finally, blastocysts were cultured and scored for three‐five days.

### Oocyte Microinjection

For microinjection, FGOs were collected and transferred to M2 medium with 5 µM milrinone‐containing (Selleck, Catalog # s2482) medium used to maintain GV arrest. Before in vitro transcript, enhanced green fluorescent protein (EGFP)‐tagged *Eif4e1b* was inserted into pcDNA3.1 eukaryote expression vector. Then, T7 mMessage mMachine kits (Invitrogen, Catalog #AM1344) were applied to synthesize 5′‐capped mRNAs (eIF4E1B‐CDS‐EGFP), and ≈10 pl of mRNAs (500 µg ml^−1^) were microinjected into the cytoplasm of FGOs.

### IF and Fluorescent Probe Detection

For ovaries, freshly isolated samples were fixed in 4% paraformaldehyde (PFA) prepared in phosphate‐buffered saline (PBS) at 4 °C overnight, and embedded in paraffin. Ovarian blocks were sectioned at a thickness of 5 µm, and processed for IHC as described before. Oocytes were isolated and fixed in 4% PFA in PBS at room temperature for half an hour, permeabilized in PBS with 0.1% Triton X‐100 for 15 min, and incubated with primary antibodies (1: 200) against eIF4E1B at 4 °C overnight. Antibody specific to 1–244aa of eIF4E1B was prepared commercially from immunizing rabbits at Shanghai Youke Biotechnology. After washing, oocytes underwent one‐hour incubation at room temperature using an Alexa Fluor 594 donkey anti‐rabbit IgG as the secondary antibody (Life Technologies, Catalog #A21207, 1:1000). At the specific stage of nascent protein synthesis, oocytes received 30‐min culturing in the medium which was free from methionine (Gibco, Catalog #21013024) and added with 50 mM methionine analog homopropargylglycine (HPG, Invitrogen, Catalog #2161847) and 1% dialyzed fetal bovine serum (FBS, 10000 MW; Sigma Aldrich). Click‐iT Cell Reaction Kit (Invitrogen, Catalog #C10429) was utilized to detect HPG. Besides, 4′,6‐diamidino‐2‐phenylindole (DAPI) was used to stain chromosomes (Sigma Aldrich, Catalog #D8417). All images were obtained under a Celldiscoverer fluorescence microscope (Zeiss LSM900, Germany) with Zen software.

### Western Blot Analysis

As previously mentioned, oocyte samples were gathered for WB analysis. The expression of corresponding proteins between different genotypes or treatments was compared by lysing oocytes in 2 × Laemmli sample buffer and loading them on the same sodium dodecyl sulfate‐polyacrylamide gel electrophoresis (SDS‐PAGE) gel. Prior to being incubated overnight at 4 °C with primary antibodies, the membranes were blocked with 5% (w/v) non‐fat dried milk powder in TBS plus 0.05% Tween 20 (TBST) at room temperature for 1 h. Next, the membrane was washed in TBST and was incubated in 5% milk‐TBST with the appropriate secondary antibody (Invitrogen, Catalog #31460/31430) for 1 h. The expression of *β*‐actin (ACTB) and DDB1 detected by anti‐*β*‐actin (ACTB) antibody (clone AC‐15; mouse monoclonal; Catalog # A1978; 1:5000) and DDB1 (rabbit polyclonal; Epitomics; Catalog #3821‐1; 1:1000) internally controlled each sample. In addition, the primary antibodies used were against eIF4E (Medical & Biological Laboratories; Catalog #RN001P; 1: 1000), CPEB1 (rabbit polyclonal; Proteintech; Catalog # 13274‐1‐AP; 1: 1000), HA (rabbit monoclonal; Sigma Aldrich; Catalog # 26183; 1: 1000), FLAG (mouse monoclonal; Sigma Aldrich; Catalog # F1804; 1: 1000), CD9 (rat polyclonal; Santa Cruz; catalog Catalog # SC18869; 1: 1000), GFP (rabbit monoclonal; Abcam; Catalog # ab32146; 1: 300) and WEE2 (rabbit polyclonal; Novus Biologicals; Catalog # NBP1‐83676; 1: 500).

### Cell Culture, Plasmid Transfection, and Co‐Immunoprecipitation

Dulbecco's MEM (DMEM, Gibco, Catalog # 11995065) added with 100 U of penicillin‐streptomycin and 10% FBS (Gibco, Catalog # 16000044) was used to cultivate 293T cells at 37 °C in 5% CO2. HA‐tagged *Ybx2*, *Igf2bp2*, *Pabpc1l*, as well as FLAG‐tagged *Eif4e1b*, were inserted into pcDNA3.0 between BamHI and XbaI. Mouse *Eif4e1b*, *Ybx2*, *Igf2bp2*, and *Pabpc1l* cDNAs were PCR‐amplified from mouse oocytes and then cloned into the eukaryote expression vector pcDNA3.0. Lipofectamine 3000 (Invitrogen, Catalog #L3000‐001) was used for transient plasmid transfection.

The lysis of cells took place in NP40 lysis buffer (10 mM Tris‐Cl with pH 7.4, 100 mM NaCl, 2.5 mM MgCl_2_, 0.5% NP 40) [50 mM Tris‐HCl with pH 7.5] blended with 1 mM phenylmethanesulfonyl fluoride (PMSF) and 1× proteinase inhibitor cocktail (Sigma, Catalog # P8340) after 48 h transfection. Then, 10 µL of protein A/G magnetic beads were used to pre‐clear protein samples at 4 °C for 1 h, and the lysate went through incubation with 10 µg of anti‐FLAG or ‐HA antibody and was rotated at 4 °C overnight, followed by the 4 h treatment of samples with 50 µL of protein A/G magnetic beads. After that, 1 × NuPAGE LDS sample buffer (Invitrogen, Catalog #NP0007) was used for the 10‐min elution of HA‐ or FLAG‐tag enriched proteins at 70 °C through vortexing at 1000 rpm. Western blotting was adopted to examine the eluted proteins. The enriched bands immunoprecipitated by lysed ovaries or oocyte samples were sliced and underwent the trypsin‐digestion of trypsin for mass spectrometry (MS) analysis. Nano ultra‐high‐performance liquid chromatography‐electrospray ionization tandem MS was employed to identify the digested peptides. Raw data files were searched against the UniProt mouse database (downloaded on June 24th, 2022, containing 55 286 entries) by using MaxQuant search engine (v.2.1.0.0) and its performed Andromeda search engine, for the purpose of obtaining the protein identifications and different label‐free quantification (LFQ) values by means of the standard parameters.

### Proteomic Analysis

Fifty FGOs and MII oocytes of every genotype (WT and *Eif4e1b*‐cKO) were gathered and dissolved in 20 µL of 0.1% diaminodiphenylmethane (DDM) (Sigma‐Aldrich, Catalog # D5172) in 25 mM Triethylammonium bicarbonate (TEAB), sonicated over ice for the lysis of cells in an interval of 1 min for ten times and centrifuged at 3000 g for 3 min. The PCR tube was added with 0.3 µL of 100 mM DL‐dithiothreital (DTT) in 25 mM TEAB. Samples went through 1‐hour incubation at 75 °C for denaturation and reduction, followed by the addition of 0.5 µL of 60 mM Iodoacetamide (IAA) (Sigma‐Aldrich, Catalog # I1149) in 25 mM TEAB (Sigma‐Aldrich, Catalog # T7408) to the PCR tube. Then, the samples experienced 30‐min incubation in the darkness at room temperature for alkylation, followed by the addition of 1 µL of 0.05 µg µL^−1^ trypsin (Promega, Catalog # V5280) in 25 mM TEAB to the PCR tube. After that, samples were digested at 37 °C overnight with gentle sharking at ≈350 g.

For MS analyses, a Thermo Fisher Orbitrap Eclipse Tribrid mass spectrometer equipped with a FAIMS Pro Interface was used to conduct experiments. Digested peptides were directly loaded on a 2 cm PEPMAP trap column (ThermoFisher Scientific) for online desalting. Besides, separation was performed on a 25 cm PepMap analytical column (ThermoFisher Scientific) with 3% to 38% buffer B (80% ACN, 0.1% formic acid) for 102 min and kept at 100% buffer B for 10 min. FAIMS switched between CVs of −45 V to −65 V with 1 s cycle time. MS1 spectra were obtained in the Orbitrap (resolution: 60k; AGQ target: standard; MaxIT: Auto; RF lens: 50%; mass range: 350 to 1500). Dynamic exclusion was used for 40 s to exclude all charge states for a specified precursor. The collection of MS2 spectra was completed in the linear ion trap (isolation window: 1.6 m/z; scan rate: rapid; AGQ target: standard; MaxIT: Auto; HCD CE: 35%; data type: centroid).

Proteome Discoverer Software (version 2.5, San Jose, CA) was used to process raw files for detecting features, searching databases, and quantifying proteins/peptides. The search of MS/MS spectra was conducted against the UniProt mouse database (downloaded on June 24th, 2022, containing 55 286 entries). Methionine oxidation and N‐terminal protein acetylation were chosen as variable modifications, while the carbamidomethylation of cysteine residues was regarded as a fixed modification. Precursors and fragments had a mass tolerance of 10 ppm and 0.6 Da, respectively. The minimum and maximum peptide lengths were six and 144 amino acids, respectively. The missed cleavage allowed for every peptide was two. The filtering of proteins had a maximum false discovery rate (FDR) of 0.01. The default settings of Proteome Discoverer were other parameters that were not mentioned.

### Single‐Cell RNA‐seq Library Preparation, Sequencing, and RNA Analysis

The library preparation of single‐cell RNA‐seq and the sequencing and RNA analysis of WT and *Eif4e1b*‐cKO oocytes in different stages were carried out according to the previous work.^[^ ^53^ ^]^ To be brief, three sets of samples of each genotype were gathered, with each sample covering three oocytes or embryos in below 3 µL of lysis buffer. A BGISEQ‐500 platform (BGI‐Shenzhen, China) with 100 bp paired‐end reads was used for the sequencing of mRNA libraries, and the trimming of all reads passing the filter removed adaptor sequences and low‐quality bases using fastp (v0.20.0). Reads were aligned to the Mus musculus reference genome GRCm38 (without masking repeats) using STAR (v2.7.3a), and transcripts per million (TPM) were calculated and normalized using StringTie (v2.0.4). R package DESeq2 (filtered with *q*‐value ≤0.01 and fold‐change ≥2) was used for analyzing differentially‐expressed genes. Sample hierarchy and differentially‐expressed gene clustering were carried out using R packages hclust and pheatmap.

### LACE‐seq Method

LACE‐seq experiments were carried out according to the published protocol.^[^ ^54^ ^]^ In short, 0.1% PFA was used for the 10‐min crosslink of 100 mouse oocytes at room temperature for 10 min, followed by their quenching via 150 mM glycine.^[^ ^32^, ^54^ ^]^ The preparation of protein A/G beads observed the instructions of the manufacturer, and their coupling with either anti‐eIF4E or ‐eIF4E1B antibody took place. The lysate of oocytes was pre‐treated with RQ1 DNase and incubated with antibody‐coated beads. After extensive wash steps, the immunoprecipitated RNAs were fragmented, and experienced 3′‐dephosphorylation and linker ligation, followed by reverse transcription on‐beads. Then, first‐strand cDNAs were derived from protein A/G beads and further captured by streptavidin C1 beads for pre‐PCR and poly(A)‐tailing to produce dsDNA as the template for in vitro transcription (IVT). IVT products were purified by the removal of the DNA template using Turbo DNase and further purified by Agencourt RNA Clean beads based on the instructions of the manufacturer. Then, linear amplified RNA was transformed into cDNA and amplified by PCR with P7 and barcoded P5 index primers. Final PCR products with a size of 130–300 bp were excised from a 2% agarose gel and purified by use of a gel purification kit (Qiagen, Catalog # 28604) as per the instructions of the manufacturer. Illumina HiSeq 2500 at BerryGenomics was used for the single‐end sequencing of all LACE‐seq libraries.

### Statistical Analyses

The results were presented as means ± standard error of mean (SEM), and each experiment was performed at least three times unless stated otherwise. Two‐tailed student's t‐tests were carried out for the comparison of two groups using GraphPad Prism. P values for bioinformatic analysis were determined by the two‐tailed Wilcoxon tests using R package. A significance level of *p* <0.05, *p* < 0.01, and *p* < 0.001 were denoted by one, two, and three asterisks, respectively.

### Ethics Approval

The Institutional Animal Care and Use Committee of the Department of Laboratory Animals in Central South University (Changsha, P. R. China) (Approval number: 2021sydw0263)

## Conflict of Interest

The authors declare no conflict of interest.

## Supporting information

Supporting Information

Supplemental Table 1

Supplemental Table 2

Supplemental Table 5

Supplemental Table 6

Supplemental Movie 1

Supplemental Movie 2

## Data Availability

The data that support the findings of this study are available from the corresponding author upon reasonable request.
