# Supplementary material for: Selective Translation of Maternal mRNA by eIF4E1B Controls Oocyte to Embryo Transition
Source: Adv Sci (Weinh). 2023 Feb 8;10(11):2205500. doi: 10.1002/advs.202205500 (PMC10104655; doi:10.1002/advs.202205500)
Supplement: Supplementary file 1 — Supporting Information [file ADVS-10-2205500-s001.pdf]

ADVANCED SCIENCE
Open Access

Supporting Information

for *Adv. Sci.*, DOI 10.1002/advs.202205500

Selective Translation of Maternal mRNA by eIF4E1B Controls Oocyte to Embryo Transition

Jing Guo, Hailian Zhao, Jue Zhang, Xiangjiang Lv, Shen Zhang, Ruibao Su, Wei Zheng, Jing Dai, Fei Meng, Fei Gong, Guangxiu Lu, Yuanchao Xue and Ge Lin**

Supporting Information

Selective Translation of Maternal mRNA by eIF4E1B Controls Oocyte to Embryo Transition

Jing Guo, Hailian Zhao, Jue Zhang, Xiangjiang Lv, Shen Zhang, Ruibao Su, Wei Zheng, Jing Dai, Fei Meng, Fei Gong, Guangxiu Lu, Yuanchao Xue*, Ge Lin*

J. Guo, J. Zhang, S. Zhang, W. Zheng, J. Dai, F. Meng, F. Gong, G.-X. Lu,G. Lin
Clinical Research Center for Reproduction and Genetics in Hunan Province,
Reproductive and Genetic Hospital of CITIC-Xiangya,
Changsha 410078, China
E-mail: linggf@hotmail.com

H.-L Zhao, R.-B. Su, Y.-C. Xue
Key Laboratory of RNA Biology
Institute of Biophysics
Chinese Academy of Sciences
Beijing 100101, China
E-mail: ycxue@ibp.ac.cn

H.-L Zhao, R.-B. Su, Y.-C. Xue
University of Chinese Academy of Sciences
Beijing 100049, China

X.-J. Lv, J. Dai, F. Gong, G.-X. Lu,G. Lin
Laboratory of Reproductive and Stem Cell Engineering
NHC Key Laboratory of Human Stem Cell and Reproductive Engineering
Central South University
Changsha 410078, China

This WORD file includes:
Supplementary Figure 1 to 6
Supplementary Table 1 to 9
Supplementary Movie 1

Supplementary Figures

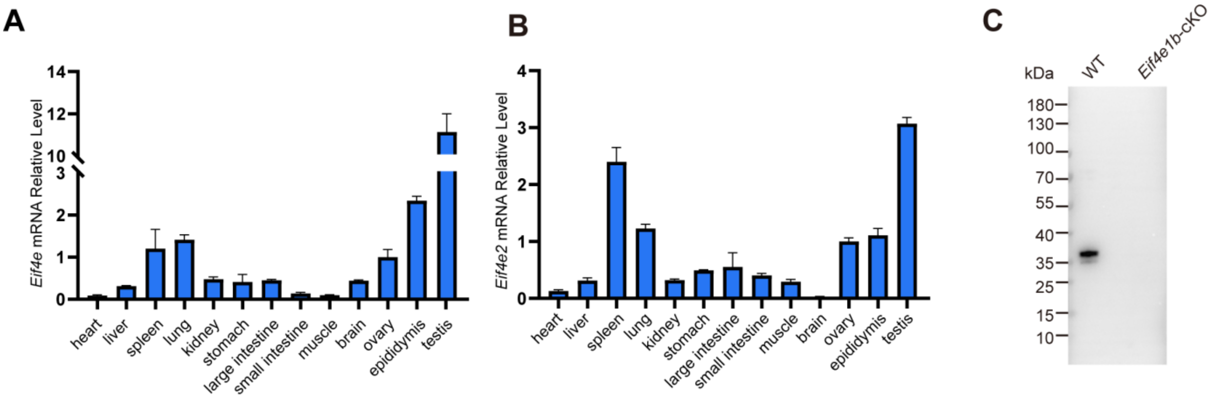

Figure S1. (A, B) Quantitative RT-PCR findings indicating relative expression levels of mouse *Eif4e* and *Eif4e2* within different tissues. **(C)** The complete film of western blotting analysis of eIF4E1B expression within WT and *Eif4e1b*-cKO GV oocytes.

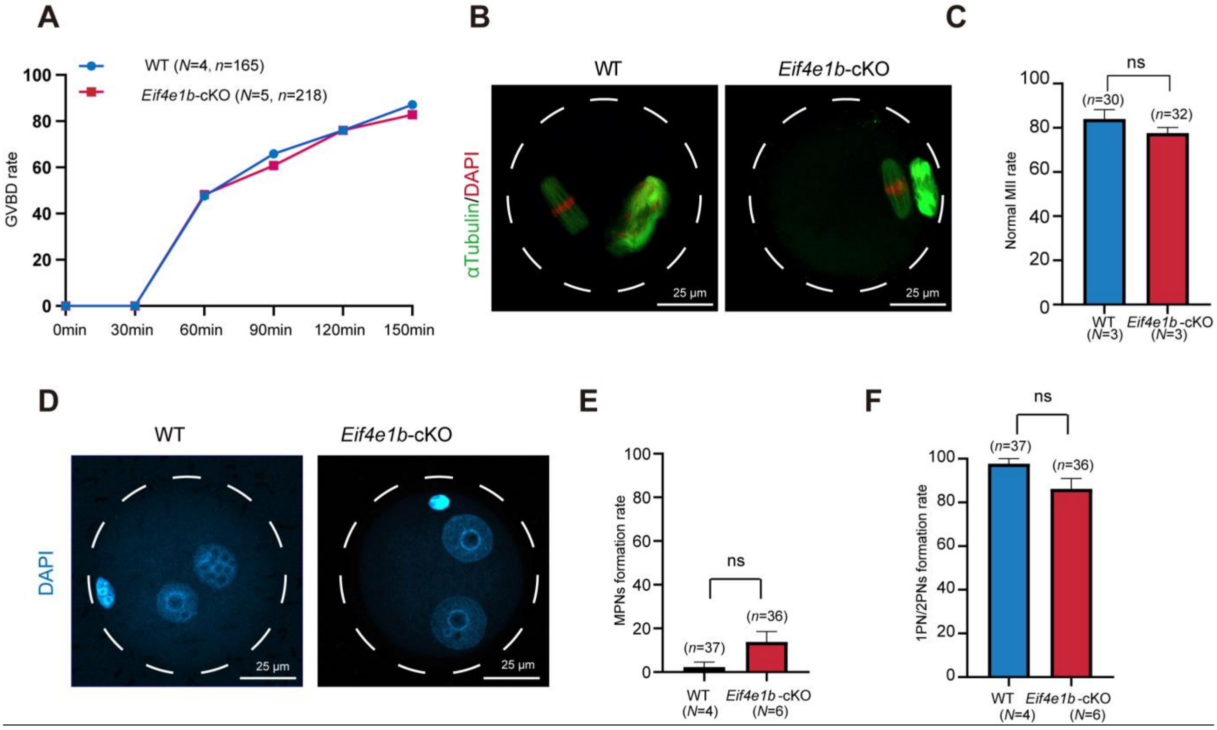

Figure S2. (A) Rates of germinal vesicle breakdown (GVBD) in WT and *Eif4e1b*-cKO GV oocytes. **(B)** Immunofluorescent images of MII oocytes from WT and *Eif4e1b*-cKO females: oocytes that are stained with α-tubulin (green) and DAPI (red). (Scale bars, 25μm) **(C)** Percentage of MII with normal morphology from WT and *Eif4e1b*-cKO females 16 h after hCG injection. **(D)** Immunofluorescent images of zygote from WT and *Eif4e1b*-cKO females: zygotes that are stained with DAPI (blue). (Scale bars, 25μm) **(E)** Percentage of zygotes with mutiple pronuclei (MPNs) and **(F)** one or two pronuclei (1PN/2PNs) from WT and *Eif4e1b*-cKO females 28 h after hCG injection. The number of analyzed oocytes is indicated (*n*).

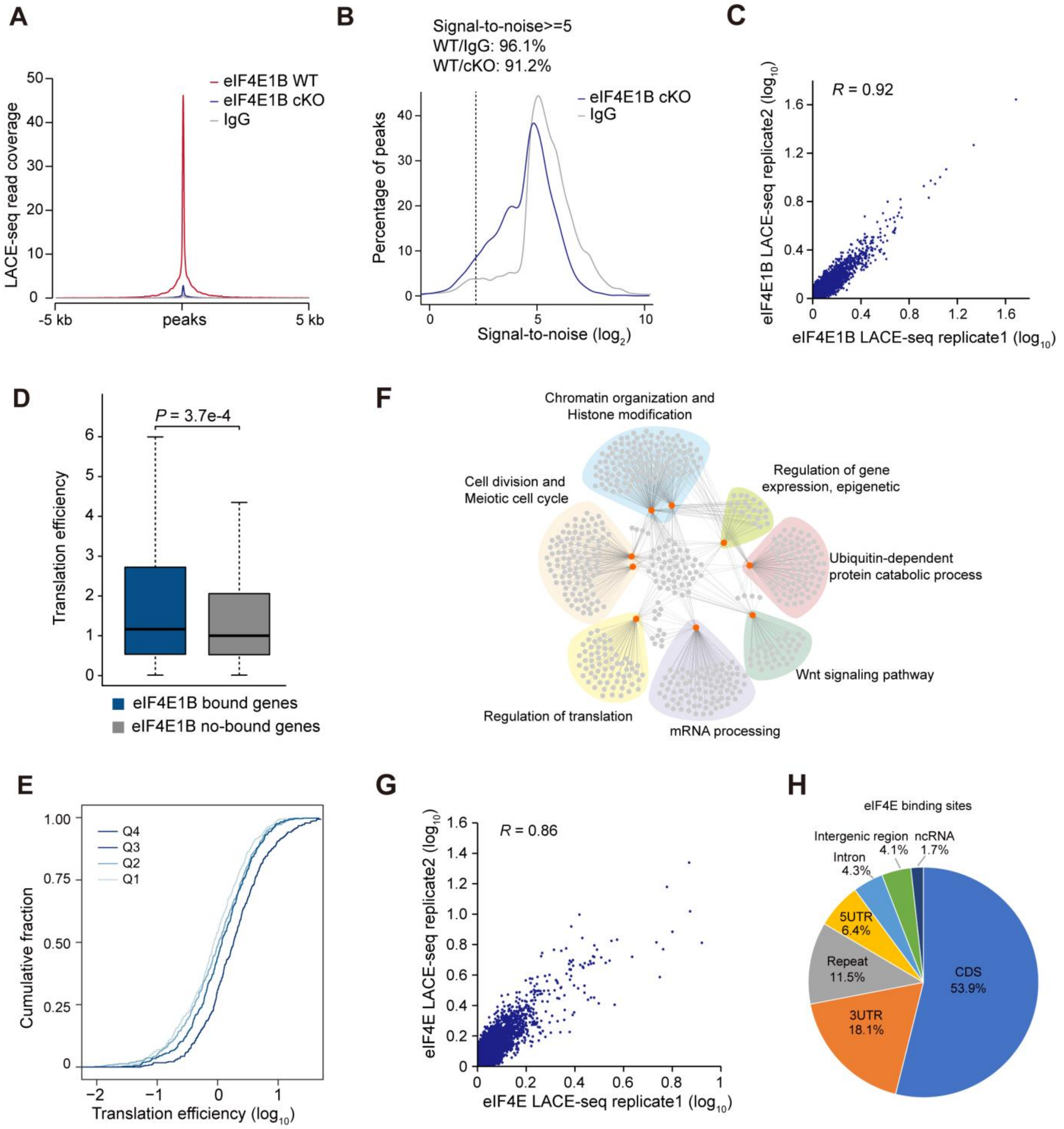

Figure S3. (A) Metaprofile of eIF4E1B LACE-seq signals from WT or cKO mouse oocytes and IgG LACE-seq around the identified peaks.**(B)** The plot showing the distribution of peaks' signal-to-noise ratio using IgG or *Eif4e1b*-cKO group as negative control. The dashed line represents the cutoff of the five-fold signal-to-noise ratio. **(C)** Scatter plot showing the reproducibility of eIF4E LACE-seq reads between two replicates. **(D)** Boxplot showing that the translation efficiency for eIF4E1B bound genes and no-bound genes. P value was determined by the two-tailed Wilcoxon test. **(E)** Cumulative distribution function (CDF) plot that presents the correlation between the eIF4E1B binding density and TE detected by Ribo-lite in GV oocytes. eIF4E1B binding RNAs involved four equal categories based on LACE-seq binding density (from low to high, Q1 to Q4, and an average of 447 RNAs for every

quarter). **(F) N**etwork analysis for enriched GO terms of *Eif4e1b* bound genes. Nodes in the network denote eIF4E1B bound genes. The individual pathways are shown in different colors. **(G)** Scatter plot showing the reproducibility of eIF4E LACE-seq reads between two replicates. *R*, Pearson correlation coefficient. (**H**) Genomic distribution of the eIF4E binding sites revealed by LACE-seq.

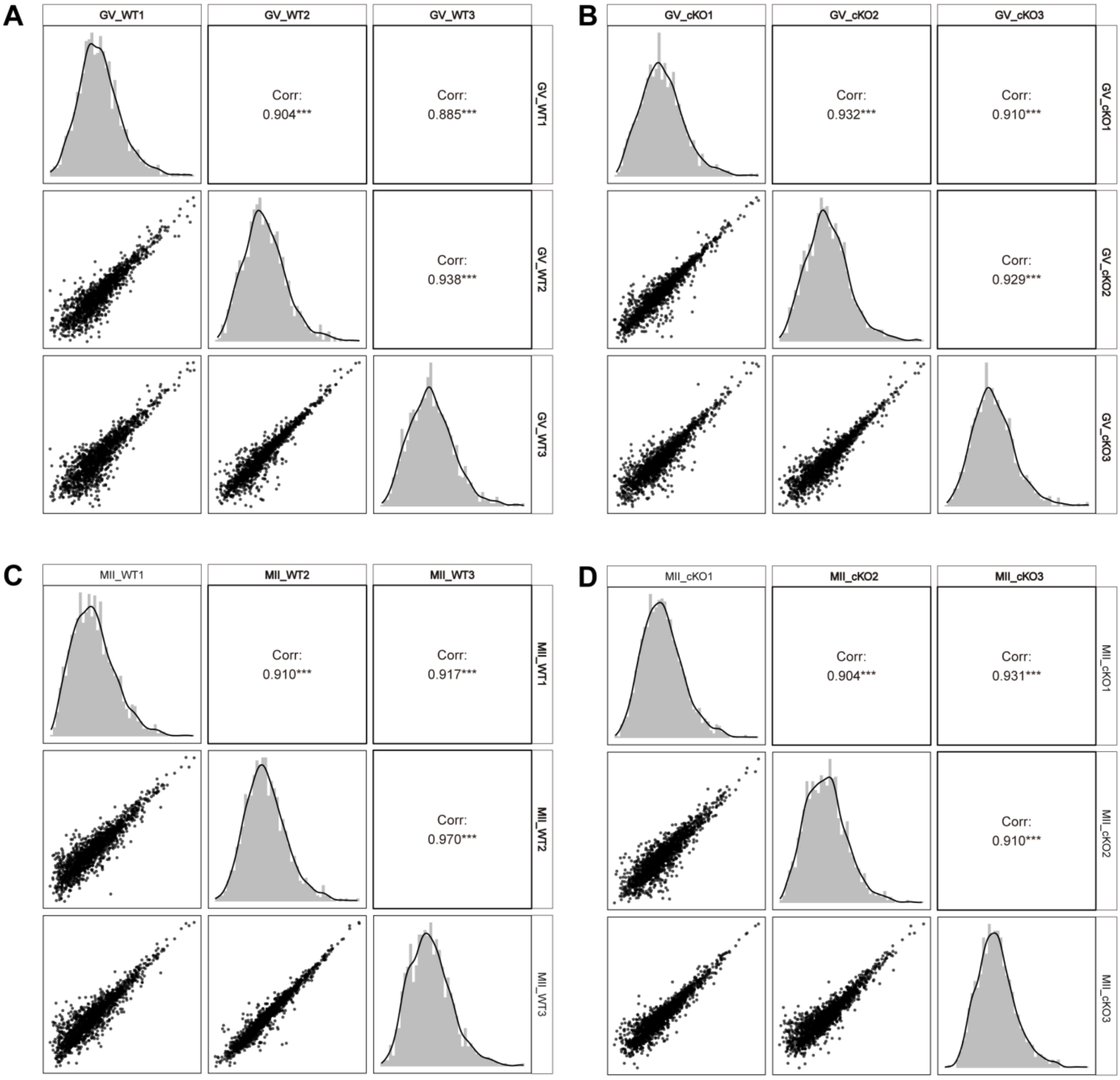

Figure S4. The mass spectrometry data were highly correlated in three biological replicates at each stage of mouse oocytes:（**A**）GV-WT; **(B)** GV-*Eif4e1b*-cKO;（**C**）MII-WT; **(D)** MII-*Eif4e1b*-cKO;

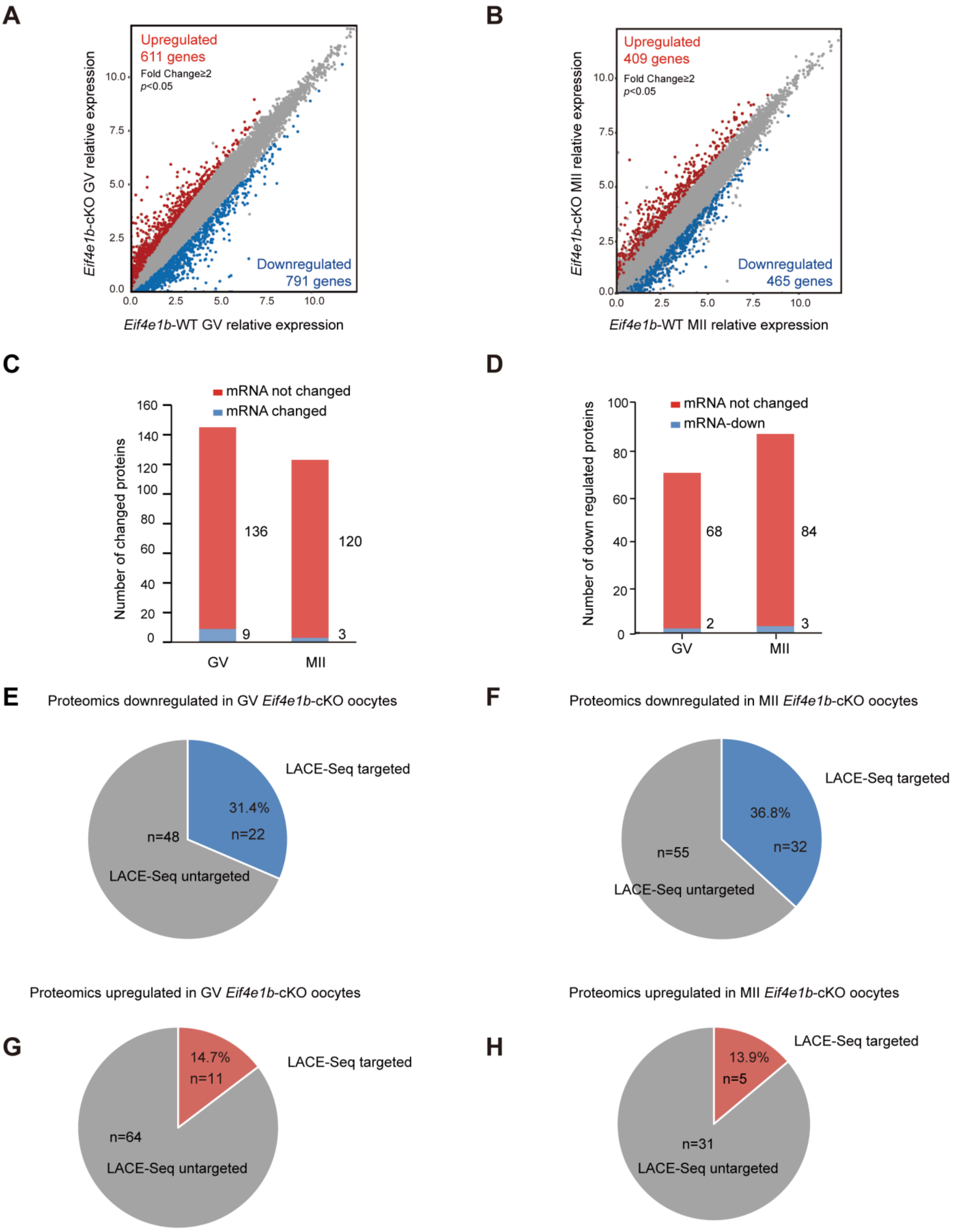

Figure S5. (A, B) Scatter plot comparing the transcripts of WT and *Eif4e1b*-cKO oocytes (fully grown GV and MII stages). Transcripts that increased or decreased by more than 2-fold in *Eif4e1b*-cKO oocytes are highlighted in red or blue, respectively **(C, D)** Proteins that are changed in GV and MII oocytes but for which the mRNA is changed or unchanged. The Thermo Fisher Orbitrap Eclipse Tribrid mass detected 1,954 protein-related transcripts used

for the analysis. **(E-H)** Venn diagrams showing the overlap of upregulated or downregulated proteins with the targets of eIF4E1B by using LACE-seq.

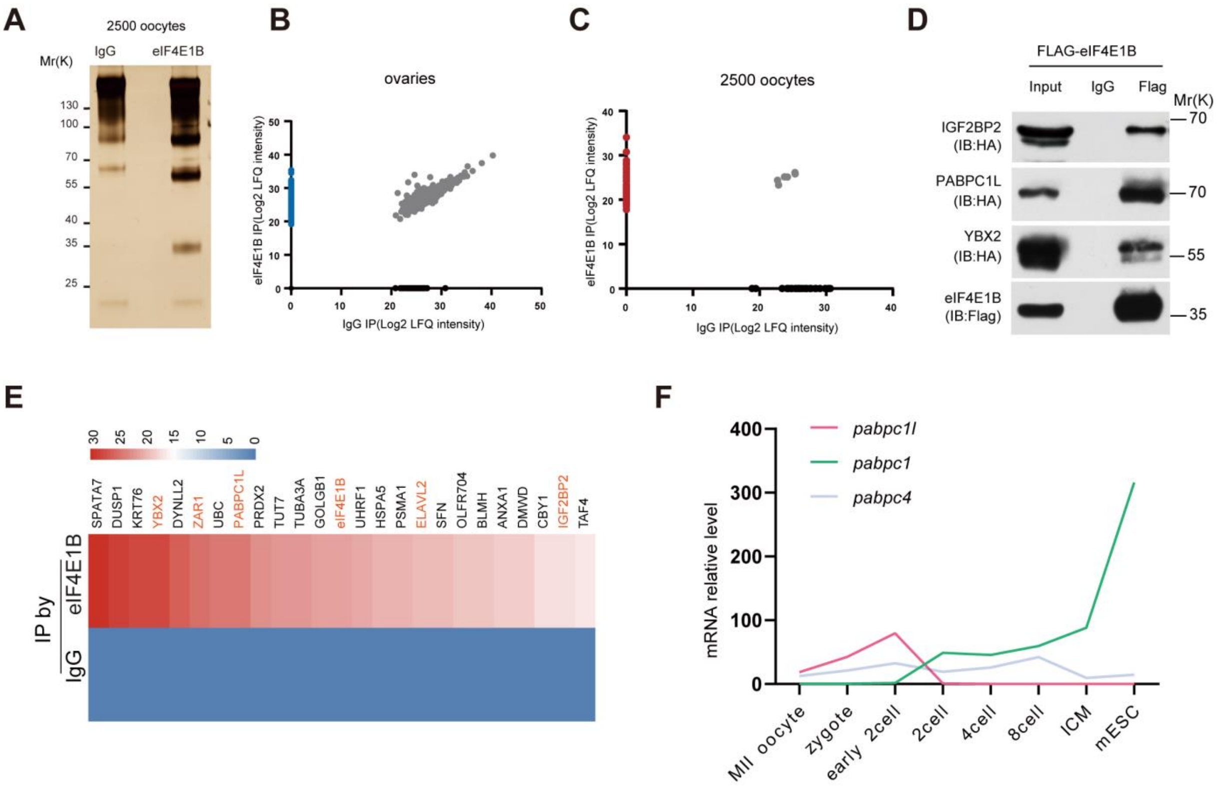

Figure S6. (A) Silver-stained gel of bound samples that are obtained from 2500 GV oocytes lysates were immunoprecipitated using IgG and ~35 kDa eIF4E1B antibody, bound proteins eluted and analysed by SDS-PAGE. **(B, C)** Mass spectrometry detected proteins from eIF4E1B IPed and control IgG IPed lysates. **(D)** CoIP assay of linkage of eIF4E1B with IGF2BP2, PABPC1L and YBX2 within co-transfected 293T cells. The FLAG-tagged eIF4E1B was co-translated with either HA-tagged IGF2BP2, PABPC1L and YBX2 in 293T cells. **(E)** Heatmap representing proteins present with different abundance within anti-eIF4E1B IPed complexes within lysis that is prepared from mouse oocytes compared with the IgG IPed negative control. The heatmap was produced through the Multi Experiment Viewer software, in which the lowest abundant proteins are in blue, the intermediate abundant proteins are in white and the highest abundant proteins are in red. **(F)** The FPKM of detected PABPs in RNA-seq data (GSE66582) at mouse MII oocytes and early embryos.

Supplementary Tables

Table S1. eIF4E1B and eIF4E targets identified by LACE-seq (Uploaded in Supporting Information)

Table S2. GO terms among eIF4E1B- (n = 1,016), eIF4E-specific targets (n = 926) and their overlapped targets (Uploaded in Supporting Information)

Table S3. Protein differentially expressed genes at GV stage

Genes	Official Full Name	LOG2(FC)
EIF4E1B	eukaryotic translation initiation factor 4E family member 1B	-11.60
USP4	ubiquitin specific peptidase 4 (proto-oncogene)	-5.58
NEFM	neurofilament, medium polypeptide	-5.51
POLM	polymerase (DNA directed), mu	-4.25
RPA1	replication protein A1	-4.11
CADM1	cell adhesion molecule 1	-3.48
GCC2	GRIP and coiled-coil domain containing 2	-3.43
OVGP1	oviductal glycoprotein 1	-3.35
TPM1	tropomyosin 1, alpha	-2.79
MAP2K4	mitogen-activated protein kinase kinase 4	-2.70
SEC31A	Sec31 homolog A (S. cerevisiae)	-2.34
D430041D05RIK	RIKEN cDNA D430041D05 gene	-2.32
ALDH16A1	aldehyde dehydrogenase 16 family, member A1	-2.15
AKR1B8	aldo-keto reductase family 1, member B8	-2.00
EIF4H	eukaryotic translation initiation factor 4H	-1.96
MRPS36	mitochondrial ribosomal protein S36	-1.92
HSP90AB1	heat shock protein 90 alpha (cytosolic), class B member 1	-1.73
DCTPP1	dCTP pyrophosphatase 1	-1.70
PSMD3	proteasome (prosome, macropain) 26S subunit, non-ATPase, 3	-1.67
EIF4E3	eukaryotic translation initiation factor 4E member 3	-1.63
HCCS	holocytochrome c synthetase	-1.57
PSMD4	proteasome (prosome, macropain) 26S subunit, non-ATPase, 4	-1.54
SUB1	SUB1 homolog, transcriptional regulator	-1.50
LAMP1	lysosomal-associated membrane protein 1	-1.45
PPP4R1	protein phosphatase 4, regulatory subunit 1	-1.43
CISD1	CDGSH iron sulfur domain 1	-1.39
CSNK2A1	casein kinase 2, alpha 1 polypeptide	-1.36
PAIP1	polyadenylate binding protein-interacting protein 1	-1.36
ADSS	adenylosuccinate synthetase, non muscle	-1.32
GAPDH	glyceraldehyde-3-phosphate dehydrogenase	-1.24
TXN1	thioredoxin 1	-1.21
YBX3	Y box protein 3	-1.19
MAN2C1	mannosidase, alpha, class 2C, member 1	-1.17
UBE2A	ubiquitin-conjugating enzyme E2A	-1.15
P4HB	prolyl 4-hydroxylase, beta polypeptide	-1.12
NLRP2	NLR family, pyrin domain containing 2	-1.05
RPL7	ribosomal protein L7	-1.04
RAB2A	RAB2A, member RAS oncogene family	-1.00
RUFY1	RUN and FYVE domain containing 1	-1.00
JPT2	Jupiter microtubule associated homolog 2	-0.99
CUL4B	cullin 4B	-0.98
RAB7	RAB7, member RAS oncogene family	-0.96
PTGES3	prostaglandin E synthase 3	-0.94

GPI1	glucose-6-phosphate isomerase 1	-0.91
DPPA5A	developmental pluripotency associated 5A	-0.89
KTN1	kinectin 1	-0.86
MFGE8	milk fat globule EGF and factor V/VIII domain containing	-0.84
PTGFRN	prostaglandin F2 receptor negative regulator	-0.72
MTUS1	mitochondrial tumor suppressor 1	-0.71
GSTP1	glutathione S-transferase, pi 1	-0.69
RPL27A	ribosomal protein L27A	-0.69
YBX2	Y box protein 2	-0.69
HSPH1	heat shock 105kDa/110kDa protein 1	-0.68
GMDS	GDP-mannose 4, 6-dehydratase	-0.67
GDI2	guanosine diphosphate (GDP) dissociation inhibitor 2	-0.67
IMMT	inner membrane protein, mitochondrial	-0.66
PRDX1	peroxiredoxin 1	-0.65
PDLIM1	PDZ and LIM domain 1 (elfin)	-0.64
ATG5	autophagy related 5	-0.63
NT5C2	5'-nucleotidase, cytosolic II	-0.61
FBXW19	F-box and WD-40 domain protein 19	-0.60
CDV3	carnitine deficiency-associated gene expressed in ventricle 3	-0.60
PLAA	phospholipase A2, activating protein	-0.55
AHCY	S-adenosylhomocysteine hydrolase	-0.55
ZP1	zona pellucida glycoprotein 1	-0.50
VAPA	vesicle-associated membrane protein, associated protein A	-0.48
HSD17B4	hydroxysteroid (17-beta) dehydrogenase 4	-0.46
KANK4	KN motif and ankyrin repeat domains 4	-0.45
TUBA1A	tubulin, alpha 1A	-0.38
TUBA1C	tubulin, alpha 1C	-0.37
CUL1	cullin 1	0.40
STRIP2	striatin interacting protein 2	0.57
CTPS	cytidine 5'-triphosphate synthase	0.57
PFN1	profilin 1	0.61
VAT1	vesicle amine transport 1	0.64
INPP5F	inositol polyphosphate-5-phosphatase F	0.64
NARS	asparaginyl-tRNA synthetase	0.71
GFUS	GDP-L-fucose synthase	0.75
CHORDC1	cysteine and histidine-rich domain (CHORD)-containing, zinc-binding protein 1	0.76
SERPINB6A	serine (or cysteine) peptidase inhibitor, clade B, member 6a	0.80
MYO10	myosin X	0.80
FDPS	farnesyl diphosphate synthetase	0.91
PSMD14	proteasome (prosome, macropain) 26S subunit, non-ATPase, 14	0.91
PAFAH1B2	platelet-activating factor acetylhydrolase, isoform 1b, subunit 2	0.93
CSE1L	chromosome segregation 1-like (S. cerevisiae)	0.96
ATOX1	antioxidant 1 copper chaperone	0.98
SARS	seryl-aminoacyl-tRNA synthetase	1.01
CSRP1	cysteine and glycine-rich protein 1	1.01
USP30	ubiquitin specific peptidase 30	1.01
SLC6A7	solute carrier family 6 (neurotransmitter transporter, L-proline), member 7	1.10
KDSR	3-ketodihydrosphingosine reductase	1.12
NLRP4E	NLR family, pyrin domain containing 4E	1.13
MTX2	metaxin 2	1.14
CAMSAP3	calmodulin regulated spectrin-associated protein family, member 3	1.19
ERMP1	endoplasmic reticulum metallopeptidase 1	1.20

SKA1	spindle and kinetochore associated complex subunit 1	1.23
BLMH	bleomycin hydrolase	1.27
SOD2	superoxide dismutase 2, mitochondrial	1.28
B4GALT2	UDP-Gal:betaGlcNAc beta 1,4- galactosyltransferase, polypeptide 2	1.28
RPA3	replication protein A3	1.28
CLIC4	chloride intracellular channel 4 (mitochondrial)	1.29
ITSN2	intersectin 2	1.33
OAS1C	2'-5' oligoadenylate synthetase 1C	1.34
CIAO1	cytosolic iron-sulfur protein assembly 1	1.34
CTSD	cathepsin D	1.35
STUB1	STIP1 homology and U-Box containing protein 1	1.36
HAT1	histone aminotransferase 1	1.38
NDUFS2	NADH:ubiquinone oxidoreductase core subunit S2	1.38
FAM114A2	family with sequence similarity 114, member A2	1.39
BSCL2	Berardinelli-Seip congenital lipodystrophy 2 (seipin)	1.41
TMEM168	transmembrane protein 168	1.55
LDHD	lactate dehydrogenase D	1.57
FBXW17	F-box and WD-40 domain protein 17	1.58
PIK3R1	phosphoinositide-3-kinase regulatory subunit 1	1.58
VPS28	vacuolar protein sorting 28	1.60
GNAS	GNAS (guanine nucleotide binding protein, alpha stimulating) complex locus	1.70
F3	coagulation factor III	1.75
SMC2	structural maintenance of chromosomes 2	1.87
TOP3B	topoisomerase (DNA) III beta	1.88
CDK5	cyclin-dependent kinase 5	1.88
FASN	fatty acid synthase	1.90
PGD	phosphogluconate dehydrogenase	1.91
GBF1	golgi-specific brefeldin A-resistance factor 1	1.93
CTNNB1	catenin (cadherin associated protein), beta 1	1.94
PDK1	pyruvate dehydrogenase kinase, isoenzyme 1	1.96
EIF4E	eukaryotic translation initiation factor 4E	2.02
PIP4K2A	phosphatidylinositol-5-phosphate 4-kinase, type II, alpha	2.03
SORL1	sortilin-related receptor, LDLR class A repeats-containing	2.05
NONE	Gm37240	2.09
CAD	carbamoyl-phosphate synthetase 2, aspartate transcarbamylase, and dihydroorotase	2.16
B3GNT2	UDP-GlcNAc:betaGal beta-1,3-N-acetylglucosaminyltransferase 2	2.19
LAMTOR3	late endosomal/lysosomal adaptor, MAPK and MTOR activator 3	2.48
PTGR1	prostaglandin reductase 1	2.53
HEXDC	hexosaminidase (glycosyl hydrolase family 20, catalytic domain) containing	2.55
SNAP91	synaptosomal-associated protein 91	2.55
PPP6R3	protein phosphatase 6, regulatory subunit 3	2.70
DBNDD1	dysbindin (dystrobrevin binding protein 1) domain containing 1	3.17
CORO7	coronin 7	3.40
SQSTM1	sequestosome 1	3.45
LARS	leucyl-tRNA synthetase	3.81
PNP	purine-nucleoside phosphorylase	3.91
MARF1	meiosis regulator and mRNA stability 1	4.61
GDAP1	ganglioside-induced differentiation-associated-protein 1	5.87
TRIM12C	tripartite motif-containing 12C	6.32
CSNK1E	casein kinase 1, epsilon	6.62

Table S4. Protein differentially expressed genes at MII stage

GENES	Official Full Name	LOG2(FC)
EIF4E1B	eukaryotic translation initiation factor 4E family member 1B	-8.15
SERPINA1C	serine (or cysteine) peptidase inhibitor, clade A, member 1C	-6.07
AZI2	5-azacytidine induced gene 2	-5.05
NDUFB11	NADH:ubiquinone oxidoreductase subunit B11	-4.37
CADM1	cell adhesion molecule 1	-4.03
PIGA	phosphatidylinositol glycan anchor biosynthesis, class A	-4.00
PRKD1	protein kinase D1	-3.81
IPO9	importin 9	-2.74
ODF2	outer dense fiber of sperm tails 2	-2.53
LPIN1	lipin 1	-2.32
VPS45	vacuolar protein sorting 45	-2.15
2210016F16RIK	RIKEN cDNA 2210016F16 gene	-2.07
ITGB5	integrin beta 5	-1.98
NRD1	nardilysin, N-arginine dibasic convertase, NRD convertase 1	-1.97
NPC1	NPC intracellular cholesterol transporter 1	-1.93
UBA2	ubiquitin-like modifier activating enzyme 2	-1.91
PGM1	phosphoglucomutase 1	-1.90
ILF2	interleukin enhancer binding factor 2	-1.72
ENTPD1	ectonucleoside triphosphate diphosphohydrolase 1	-1.71
TIPARP	TCDD-inducible poly(ADP-ribose) polymerase	-1.70
1810058I24RIK	RIKEN cDNA 1810058I24 gene	-1.64
OBOX2	oocyte specific homeobox 2	-1.61
ARHGEF7	Rho guanine nucleotide exchange factor (GEF7)	-1.61
EIF4E3	eukaryotic translation initiation factor 4E member 3	-1.60
HNRNPL	heterogeneous nuclear ribonucleoprotein L	-1.58
OBOX1	oocyte specific homeobox 1	-1.57
TRA2A	transformer 2 alpha	-1.51
NUDT14	nudix (nucleoside diphosphate linked moiety X)-type motif 14	-1.45
TUBB6	tubulin, beta 6 class V	-1.42
PCYT2	phosphate cytidylyltransferase 2, ethanolamine	-1.41
NAA50	N(alpha)-acetyltransferase 50, NatE catalytic subunit	-1.40
E330021D16RIK	RIKEN cDNA E330021D16 gene	-1.37
CCNB1	cyclin B1	-1.32
SPAG1	sperm associated antigen 1	-1.25
MAN2C1	mannosidase, alpha, class 2C, member 1	-1.22
OBOX5	oocyte specific homeobox 5	-1.21
TUBB5	tubulin, beta 5 class I	-1.19
GAPDH	glyceraldehyde-3-phosphate dehydrogenase	-1.19
HSP90AB1	heat shock protein 90 alpha (cytosolic), class B member 1	-1.17
MAP2K4	mitogen-activated protein kinase kinase 4	-1.17
PRC1	protein regulator of cytokinesis 1	-1.16
SLC26A6	solute carrier family 26, member 6	-1.14
GSR	glutathione reductase	-1.12
FAM98C	family with sequence similarity 98, member C	-1.09
RHBDF2	rhomboid 5 homolog 2	-1.07
ABCF3	ATP-binding cassette, sub-family F (GCN20), member 3	-1.07
ITGA6	integrin alpha 6	-1.04
CLCC1	chloride channel CLIC-like 1	-1.04
ACLY	ATP citrate lyase	-1.03
ITGB1	integrin beta 1 (fibronectin receptor beta)	-1.01

NAALAD2	N-acetylated alpha-linked acidic dipeptidase 2	-1.01
PTGFRN	prostaglandin F2 receptor negative regulator	-1.00
SAE1	SUMO1 activating enzyme subunit 1	-0.97
ACOT7	acyl-CoA thioesterase 7	-0.95
PTGES3	prostaglandin E synthase 3	-0.93
PFKL	phosphofructokinase, liver, B-type	-0.93
ARG2	arginase type II	-0.92
AKAP12	A kinase (PRKA) anchor protein (gravin) 12	-0.91
ITIH2	inter-alpha trypsin inhibitor, heavy chain 2	-0.90
FBXW19	F-box and WD-40 domain protein 19	-0.90
CSAD	cysteine sulfinic acid decarboxylase	-0.88
GBE1	glucan (1,4-alpha-), branching enzyme 1	-0.88
CPSF1	cleavage and polyadenylation specific factor 1	-0.87
VDAC1	voltage-dependent anion channel 1	-0.87
OSBPL8	oxysterol binding protein-like 8	-0.83
MINDY3	MINDY lysine 48 deubiquitinase 3	-0.81
PPM1H	protein phosphatase 1H (PP2C domain containing)	-0.78
CUL3	cullin 3	-0.75
ZAR1	zygote arrest 1	-0.74
MYH11	myosin, heavy polypeptide 11, smooth muscle	-0.74
DIPK2A	divergent protein kinase domain 2A	-0.73
CSNK1A1	casein kinase 1, alpha 1	-0.73
FUBP1	far upstream element (FUSE) binding protein 1	-0.73
ABHD12	abhydrolase domain containing 12	-0.71
MAP4	microtubule-associated protein 4	-0.68
TACC3	transforming, acidic coiled-coil containing protein 3	-0.67
FIP1L1	FIP1 like 1 (S. cerevisiae)	-0.61
RNF25	ring finger protein 25	-0.60
ADK	adenosine kinase	-0.55
KIF2A	kinesin family member 2A	-0.46
PANK2	pantothenate kinase 2	-0.45
CKB	creatine kinase, brain	-0.43
LMNA	lamin A	-0.40
TACC2	transforming, acidic coiled-coil containing protein 2	-0.40
OGFOD2	2-oxoglutarate and iron-dependent oxygenase domain containing 2	-0.39
TUBB4B	tubulin, beta 4B class IVB	-0.39
USO1	USO1 vesicle docking factor	-0.24
NSF	N-ethylmaleimide sensitive fusion protein	0.31
AARS	alanyl-tRNA synthetase	0.35
PCCA	propionyl-Coenzyme A carboxylase, alpha polypeptide	0.40
ATP8A2	ATPase, aminophospholipid transporter-like, class I, type 8A, member 2	0.40
ELOB	elongin B	0.41
PSMD5	proteasome (prosome, macropain) 26S subunit, non-ATPase, 5	0.44
LAMTOR3	late endosomal/lysosomal adaptor, MAPK and MTOR activator 3	0.49
GLMN	glomulin, FKBP associated protein	0.60
CBR1	carbonyl reductase 1	0.67
ACADS	acyl-Coenzyme A dehydrogenase, short chain	0.78
STAT3	signal transducer and activator of transcription 3	0.80
ACO2	aconitase 2, mitochondrial	0.80
ARMC10	armadillo repeat containing 10	0.81
F3	coagulation factor III	0.84
LSM8	LSM8 homolog, U6 small nuclear RNA associated	0.85

ADA	adenosine deaminase	0.87
OXCT1	3-oxoacid CoA transferase 1	1.00
EIF4E	eukaryotic translation initiation factor 4E	1.05
LAMB1	laminin B1	1.10
ACOT13	acyl-CoA thioesterase 13	1.25
CTSD	cathepsin D	1.47
SCRN2	secernin 2	1.65
NCL	nucleolin	1.75
PNP	purine-nucleoside phosphorylase	1.82
RAB11FIP5	RAB11 family interacting protein 5 (class I)	1.89
PEPD	peptidase D	1.90
PFKP	phosphofructokinase, platelet	2.05
ACSF2	acyl-CoA synthetase family member 2	2.08
SNAP91	synaptosomal-associated protein 91	2.08
ETFDH	electron transferring flavoprotein, dehydrogenase	2.35
SQSTM1	sequestosome 1	2.38
GM20431	Gm20431	3.55
HINT3	histidine triad nucleotide binding protein 3	3.94
GDI1	guanosine diphosphate (GDP) dissociation inhibitor 1	3.95
GTPBP1	GTP binding protein 1	5.10
PDCD10	programmed cell death 10	6.32

Table S5. The InterPro domain analysis of eIF4E1B-interacting proteins (Uploaded in Supporting Information)

Table S6. The poly A length of RNAs in GV and MII oocytes (Uploaded in Supporting Information)

Table S7. Genotyping Primer information

Name	Species	Sequence	Product size(bp)
Eif4e1b-Flox-F	Mouse	TATTAGGCTGCTGCCTTAGTGTG	Homozygotes:204 bp<br>Heterozygotes:204 bp and 137 bp<br>Wildtype allele:137 bp
Eif4e1b-Flox-R	Mouse	TTCTCTGGGAACTTGTCTGAACTA	
Zp3-Cre-F	Mouse	GAACGCACTGATTTCGACCA	Targeted: 430 bp
Zp3-Cre-R	Mouse	GCTAACCAGCGTTTTCGTTC	

Table S8. Primer information

Name	Gene Bank ID	RNA ID	Species	Sequence	Product size(bp)
Eif4e1b-F	218268	NM_001033269.3	Mouse	AGTGATGCAGATCGGGCAAA	133bp
Eif4e1b-R			Mouse	CCGGTCTCACACCACAAACT	

Eif4e-F	13684	NM_007917.4	Mouse	ACCCCTACCACTAATCCCCC	162bp
Eif4e-R			Mouse	CAATCGAAGGTTTGCTTGCCA	
Eif4e2-F	26987	NM_001039170.1	Mouse	CTCATCCTGGCTATGCTCGG	225bp
Eif4e2-R			Mouse	GCCTGGCATTTTGATGCTGT	

Table S9. The cited published datasets

**Title**	**Journal (year)**	**Paper website**	**License and accessibility**
Ultrasensitive Ribo-seq reveals translational landscapes during mammalian oocyte-to-embryo transition and pre-implantation development	Nature Cell Biology (2022)	https://www.nature.com/articles/s41556-022-00928-6	GSE165782
Allelic reprogramming of the histone modification H3K4me3 in early mammalian development	Nature (2016)	https://www.nature.com/articles/nature19361	GSE71434
The landscape of accessible chromatin in mammalian pre-implantation embryos (RNA-Seq)	Nature (2016)	https://www.nature.com/articles/nature18606	GSE66582

Supplementary Movie 1 (Uploaded in Supporting Information)
Movie S1-A, B: (A) MII oocytes recovered from WT females IVF with normal sperms; **(B)**Accumulation of multiple spermatozoa in the perivitelline space of the eggs recovered from *Eif4e1b*-cKO females IVF with normal sperms.
